# Dynamic formation and transcriptional regulation mediated by phytohormones during chalkiness formation in rice

**DOI:** 10.1186/s12870-021-03109-z

**Published:** 2021-06-30

**Authors:** Qin Xie, Jinke Xu, Ke Huang, Yi Su, Jianhua Tong, Zhigang Huang, Chao Huang, Manlin Wei, Wanhuang Lin, Langtao Xiao

**Affiliations:** 1 Hunan Provincial Key Laboratory of Phytohormones and Growth Development, Hunan Agricultural University, Changsha, 410128 China; 2 College of Bioscience and Biotechnology, Hunan Agricultural University, Changsha, 410128 China

**Keywords:** Rice, Chalkiness, Dynamic formation, Phytohormones, Transcriptional regulation

## Abstract

**Background:**

Rice (*Oryza sativa* L.) Chalkiness, the opaque part in the kernel endosperm formed by loosely piled starch and protein bodies. Chalkiness is a complex quantitative trait regulated by multiple genes and various environmental factors. Phytohormones play important roles in the regulation of chalkiness formation but the underlying molecular mechanism is still unclear at present.

**Results:**

In this research, Xiangzaoxian24 (X24, pure line of *indica* rice with high-chalkiness) and its origin parents Xiangzaoxian11 (X11, female parent, pure line of *indica* rice with high-chalkiness) and Xiangzaoxian7 (X7, male parent, pure line of *indica* rice with low-chalkiness) were used as materials. The phenotype, physiological and biochemical traits combined with transcriptome analysis were conducted to illustrate the dynamic process and transcriptional regulation of rice chalkiness formation. Impressively, phytohormonal contents and multiple phytohormonal signals were significantly different in chalky caryopsis, suggesting the involvement of phytohormones, particularly ABA and auxin, in the regulation of rice chalkiness formation, through the interaction of multiple transcription factors and their downstream regulators.

**Conclusion:**

These results indicated that chalkiness formation is a dynamic process associated with multiple genes, forming a complex regulatory network in which phytohormones play important roles. These results provided informative clues for illustrating the regulatory mechanisms of chalkiness formation in rice.

**Supplementary Information:**

The online version contains supplementary material available at 10.1186/s12870-021-03109-z.

## Background

Chalkiness, a negative trait for rice quality, represents the white/opaque part in rice (*Oryza sativa* L.) endosperm. Rice chalkiness is tightly correlated to the cooking performance and thus is a key concerned feature in rice market [1]. Due to the loose starch structure, chalkiness is usually presented in white, thus chalkiness can be classified into white-belly, white-core, white-base, white-back and milky-white types according to the location. Among them, white-core and white-belly are the most common chalky types [2]. Scanning electron microscope (SEM) study revealed that the opaque part is loosely packed with starch granules and protein bodies (PBs), which make the grain vulnerable to cracking during the polishing process [3].

Chalkiness is a complex trait controlled by quantitative trait loci (QTLs). Multiple QTLs contributing to grain chalkiness have been mapped across all 12 chromosomes of the rice genome [4]. Two QTLs controlling the percentage of grains with chalkiness (PGWC), *qPGWC-7* [5] and *qPGWC-9* [6], are located on chromosomes 7 and 9 respectively. As a major QTL for grain width (GW), *GW2* significantly increases percentage of chalky rice as well as grain width and weight [7]. Being a QTL for the percentage of chalky grains (PCG), *qPCG1* is located in a 139 kb region on the long arm of chromosome 1 [8]. In our previous research, 4 QTLs (*chal1*, *chal2*, c*hal3* and *chal4*) associated with chalkiness were respectively mapped on chromosomes 2 and 6 [9]. However, the research progress is still relatively slow in the genetic foundation of chalkiness. Although several chalkiness related QTLs and genes were isolated and functionally analyzed, the formation and regulation mechanism of rice chalkiness is far from clear [10, 11].

Chalkiness formation is also influenced by various environmental factors. The poor environmental conditions of high temperature and drought stress strongly promote chalkiness formation. At the grain filling stage, high temperature stress could inhibit the expression of the starch synthesis genes, such as *GBSSI* and *BEs*, reducing amylose content and increasing long chain amylopectin [12, 13]. Under high temperature stress, the up-regulated expression of α-amylase genes (e.g. *Amy1C*, *Amy3A*, *Amy3D* and *Amy3E*) in the endosperm of rice grains could enhance the starch degradation and chalkiness formation [14]. Drought stress could induce the expression of antioxidant enzyme related genes followed by the increase of sucrose synthase, which would lead to chalkiness formation [15, 16]. In addition, the decreased photosynthetic products under the insufficient sunlight, and shortened grain filling time under the excessive sunlight exposure could result in increasing chalkiness [17]. Generally, high temperature, drought and excessive or insufficient sunlight mainly promote the rice chalkiness formation due to the abnormal expression of carbon metabolism-related genes [18–21].

At present, it is generally acknowledged that the rice chalkiness is the result of insufficient starch synthesis or excess degradation followed by loose starch granules. Mutations in some starch synthesis genes, such as *Waxy* [22], *SSIIIa* [23], *BEIIb* [24], *OsAPL2* [25] and *OsPPDKB* [26], resulted in a chalky phenotype. Moreover, other amyloplast development related factors, such as *FLO*2 [27], *FLO6* [28], *OsPho1* [29], and *ISA1* [30], play important roles in starch accumulation and chalkiness formation. Storage protein is the second largest component in endosperm, and thus protein metabolism also affects chalkiness formation. Prolamins and globulins were found to be significantly lower in the chalky part than that in non-chalkiness part [31]. Expression of two genes encoding 13 kDa prolamin decreased across all developmental stages in the chalky part of a notched-belly rice mutant [3], and both genes were also down-regulated in the chalky grains caused by high temperature [18, 32].

In addition, multiple complex regulatory pathways are involved in chalkiness formation. Transcription factors NF-YB1 [33, 34], RSR1 [35] and OsbZIP58 [36] were reported to be involved in chalkiness formation. *OsCDPK1* was reported to affect the physicochemical properties of starch and plays key roles in negatively controlling the grain size, amylose content, and endosperm appearance [37]. *Chalk5* [10], *PDIL1-1* [38], *BiP1* [39, 40], *OsVPS9A* [41], *OsRAB5A* [42] and *GPA3* [43] involved in protein metabolism were reported to mediate chalkiness formation.

Recently, it was found that phytohormones are involved in chalkiness formation. Higher auxin, cytokinins (CKs) and gibberellins (GAs) levels might result in more chalkiness, while brassionosteroids (BRs) could reduce chalkiness [44]. Our previous research revealed that increased level of ABA during early to middle grain filling stage caused by high temperature was more responsible for chalkiness formation [45]. The ABA/GA ratio was significantly correlated with grain filling, high ABA/GA ratio could promote the grain filling and reduce chalkiness [46]. However, the regulatory network mediated by phytohormones is still unknown.

In this study, 3 rice cultivars with stable chalkiness phenotype were employed as the experimental materials to study transcriptional regulation during chalkiness formation, i.e. Xiangzaoxian24 (X24, pure line of *indica* rice with high-chalkiness) and its origin parents Xiangzaoxian11 (X11, female parent, pure line of *indica* rice with high-chalkiness) and Xiangzaoxian7 (X7, male parent, pure line of *indica* rice with low-chalkiness). The phenotype, physiological and biochemical traits combined with transcriptome sequencing were analyzed in caryopsis. The results indicated that many genes involved in starch/sucrose/protein metabolism, transcriptional regulation and kinase signals were differentially expressed between high (X11 and X24) and low (X7) chalkiness caryopsis. Further analysis found that phytohormones might mediate the rice chalkiness formation through the interactions of transcription factors and other regulators. Accordingly, chalkiness formation is a dynamic process associated with multiple genes, and is regulated by a complex regulatory network, in which phytohormones play a crucial role. Our research would provide informative clues for illustrating the regulatory mechanisms of chalkiness formation in rice.

## Results

### Characterization of X24 and its two origin parental lines

**Fig. 1:**
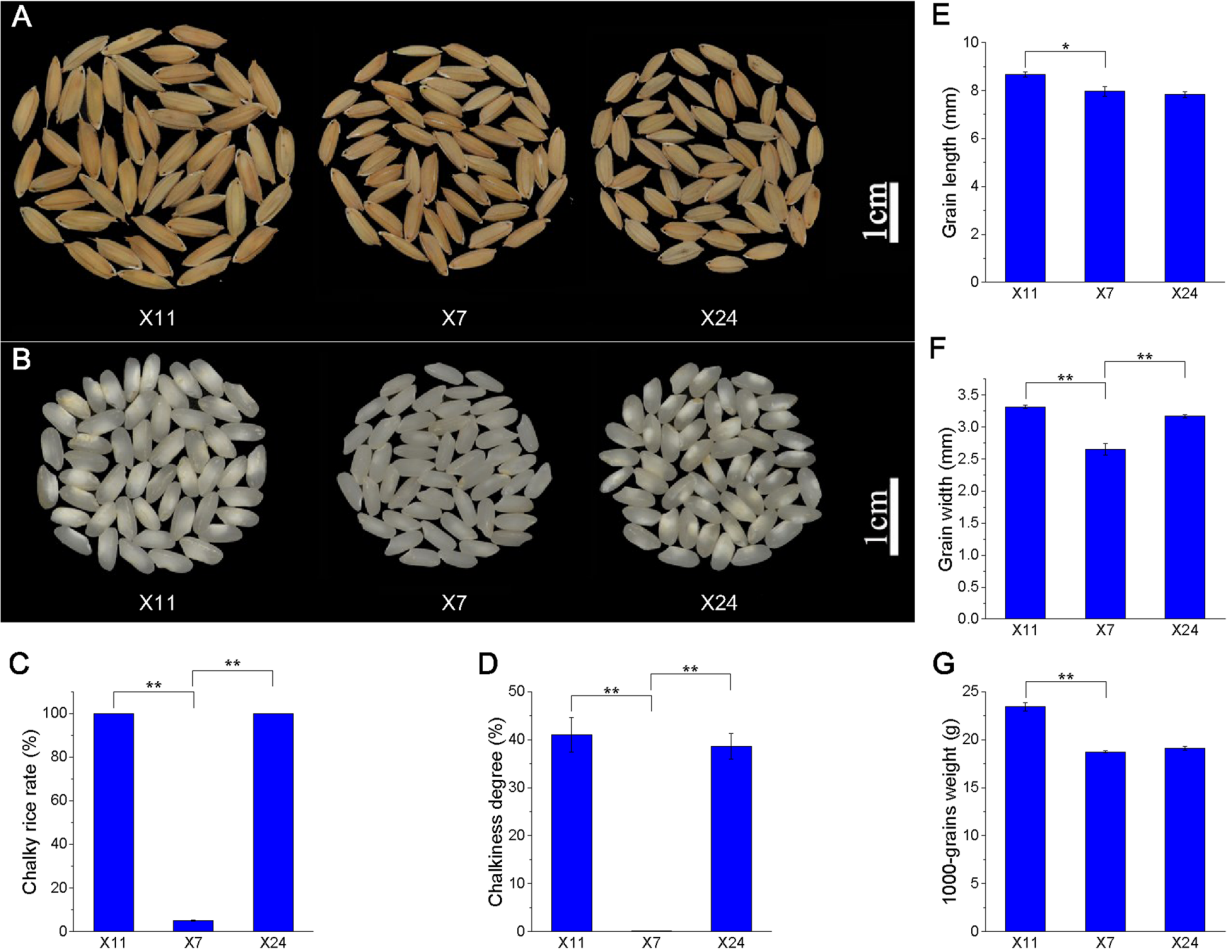
The phenotype of mature grains (**A**), chalkiness trait (**B**), chalky rice rate (**C**), chalkiness degree (**D**), grain length (**E**), grain width (**F**) and 1000-grains weight (**G**) of X11, X7 and X24. Data shown as means ± SD of three biological replicates (n = 30). Asterisks indicate a significant difference based on a Dunnett’s test. * significant difference at 5% level (*P* < 0.05); ** significant difference at 1% level (*P* < 0.01)

X24 (pure line of *indica* rice with high-chalkiness) and its origin parents X11 (female parent, pure line of *indica* rice with high-chalkiness) and X7 (male parent, pure line of *indica* rice with low-chalkiness) are widely planted in Hunan Province. The full growth stage of X11, X7 and X24 were 108–109 days, 108–110 days and 105–108 days; the average plant height of X11, X7 and X24 were 80 cm, 75 cm and 74.2 cm; the yield of X11, X7 and X24 were about 7210 kg/ha, 6300 kg/ha and 6618 kg/ha, respectively [47]. The phenotypes of mature grain and chalkiness trait were shown in Fig. 1A-B. The mature rice grains of X11 and X24 showed 100% chalky rice rate with 40.99% and 38.60% chalkiness degree respectively, while X7 showed 5% chalky rice rate with 0.127% chalkiness degree (Fig. 1C-D). The grain length of X11, X7 and X24 were 8.67 mm, 7.97 mm and 7.83 mm; the grain width of X11, X7 and X24 were 3.31 mm, 2.65 mm and 3.17 mm; the 1000-grains weight of X11, X7 and X24 were 23.44 g, 18.73 g and 19.12 g, respectively (Fig. 1E-G). The stable chalkiness phenotype of X11 and X24 showed significant differences from X7. Therefore, these rice cultivars were proper materials for studying the rice chalkiness formation and its regulation at transcription level.

### Dynamic formation of chalkiness at grain filling stage

**Fig. 2:**
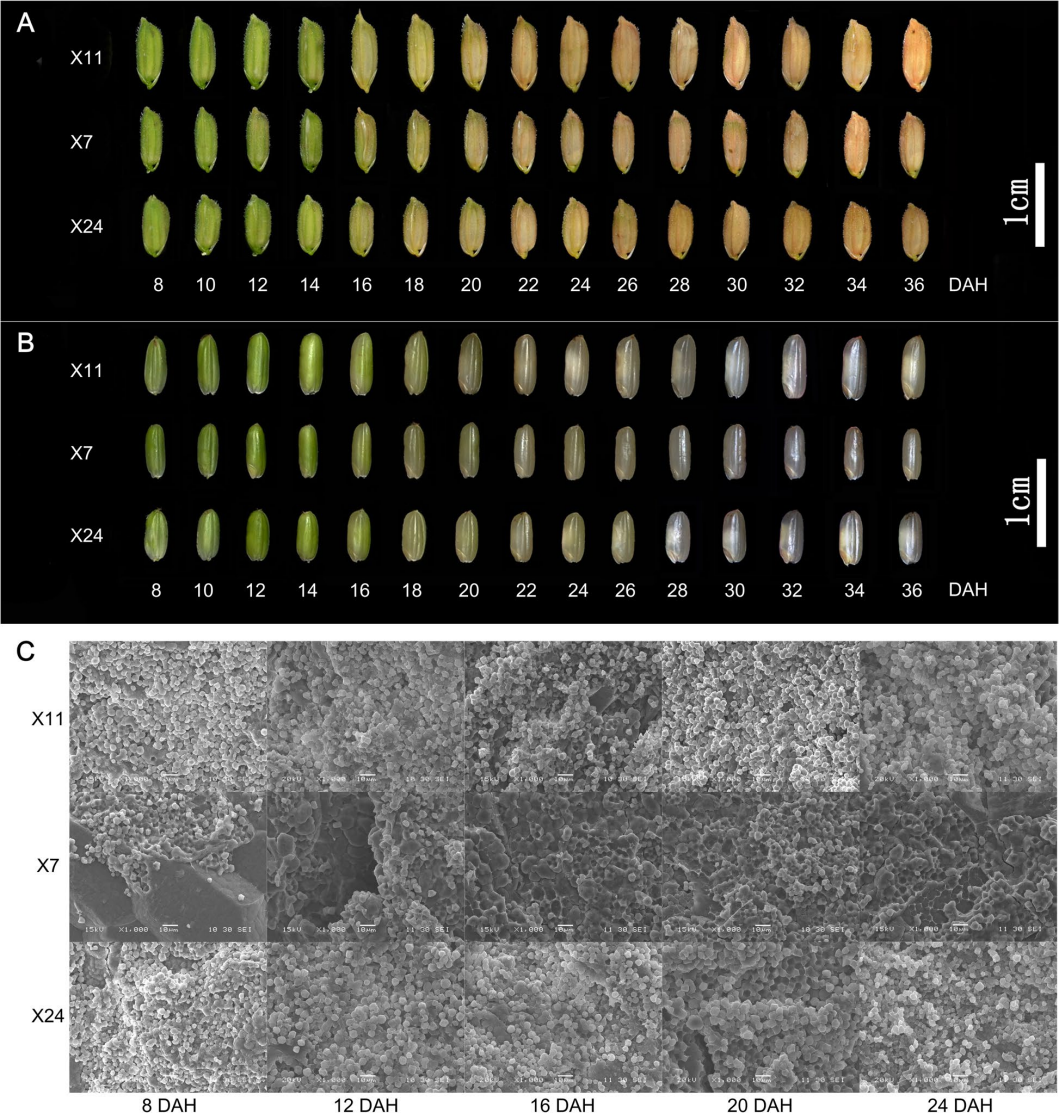
The dynamics of grain filling and chalkiness formation traits (**A** and **B**). The starch granules in endosperms examined by SEM at 8 DAH, 12 DAH, 16 DAH, 20 DAH and 24 DAH (Scale bars: 10 μm) (**C**)

Rice caryopses were collected to analyze the dynamics of grain filling and chalkiness formation every 2 days from 8 to 36 days after heading (DAH). The glumes of caryopsis were green from 8 to 14 DAH, and the color of glumes changed from green to yellow since 16 DAH until mature (Fig. 2A). No significant differences were found in color changes of glumes among X11, X24 and X7 at grain filling stage. The caryopsis was still green from 8 to 12 DAH, but from 20 DAH, the caryopsis of X7 began to translucent, while the caryopsis of X11 and X24 began to emerge chalkiness with opaque at belly of caryopsis (Fig. 2B). The caryopsis at 8 DAH were filled with diluted white milky, and the caryopsis began to harden at 12 DAH filling with concentrated white milky. As the continuous accumulation of starch and protein, the dry and fresh weight of caryopsis continued to increase, and the caryopsis was gradually hardened since 16 DAH. Along with the grain filling, the caryopsis of X7 was translucent, while the chalkiness trait of X11 and X24 became more obvious.

The caryopses collected at 8 DAH, 12 DAH, 16 DAH, 20 DAH and 24 DAH were respectively observed by SEM in order to examine whether the morphology of the endosperm starch granules was different between high and low chalkiness caryopsis. SEM examinations showed that endosperms of X11 and X24 carried round, small and loosely packed starch granules with air spaces, while endosperms of X7 were filled with large and tightly packed starch granules at 8 DAH. The results of SEM at 12 DAH, 16 DAH, 20 DAH and 24 DAH were similar to that at 8 DAH (Fig. 2C). Since 8 DAH, a large amount of starch and storage protein accumulated rapidly in endosperm. Therefore, we speculated that the genes regulating starch and storage protein metabolism were differentially expressed between high and low chalkiness caryopsis. The size, shape and arrangement of starch granules were different between high and low chalkiness caryopsis, which eventually led to chalkiness formation. The results of phenotypic observation and SEM indicated that 8 DAH, 12 DAH and 16 DAH were the three critical periods at grain filling stage. Thus we sampled at 8 DAH, 12 DAH and 16 DAH for phytohormonal determination and transcriptome analysis.

### Analysis of DEGs in caryopsis

**Fig. 3:**
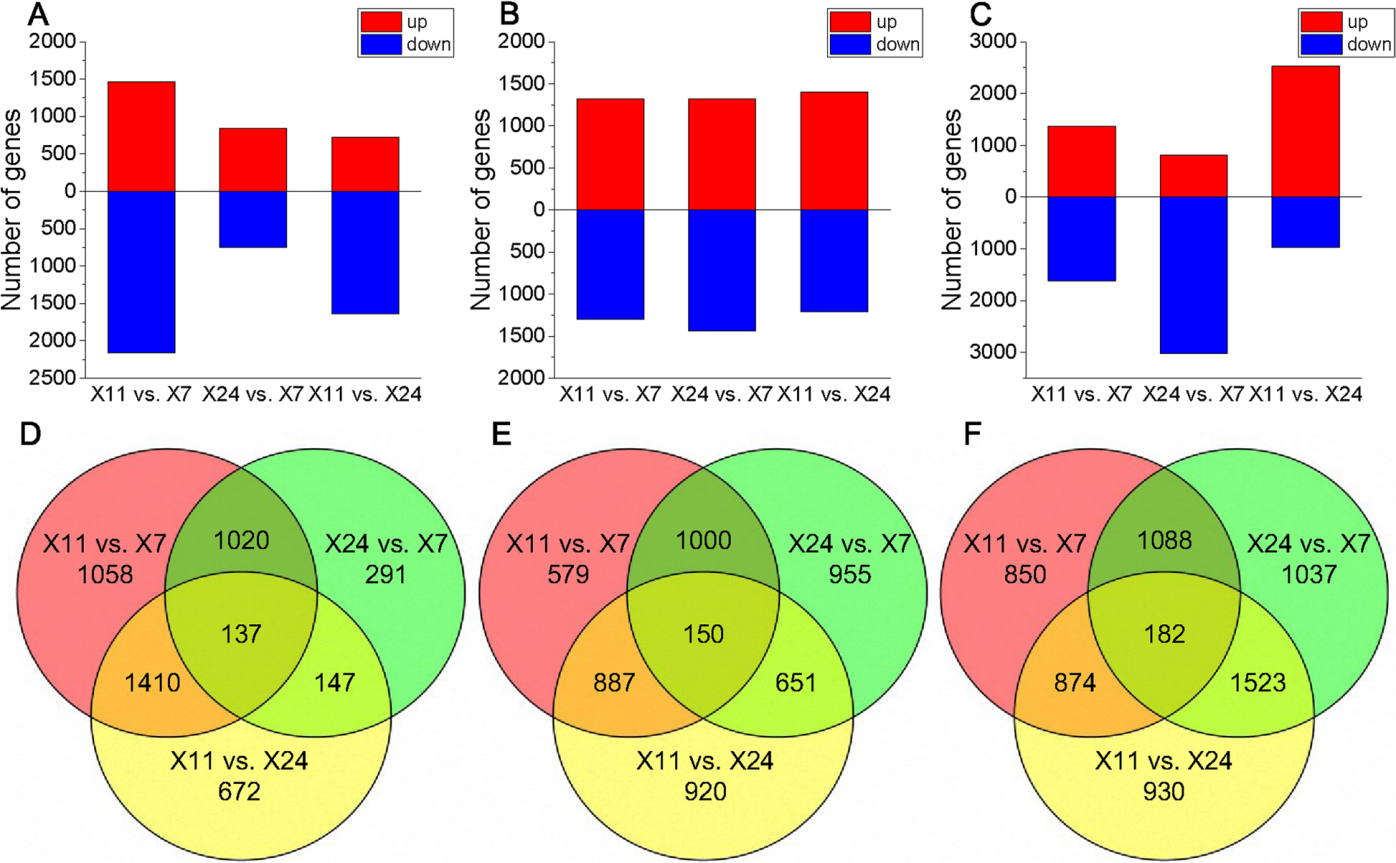
DEGs obtained by X11 vs. X7, X24 vs. X7 and X11 vs. X24 at (**A**) 8 DAH, (**B**) 12 DAH and (**C**) 16 DAH. Red column represents up-regulated of genes, blue column represents down-regulated of genes. Venn diagram of DEGs among X11, X7 and X24 at (**D**) 8 DAH, (**E**) 12 DAH and (**F**) 16 DAH. DEGs were differentially expressed with statistical significance (*P*-value ≤ 0.05 and |Log_2_foldchange(FC)|≥ 1)

The gene expression was estimated using the fragments per kilobase per million (FPKM) method. Putative differentially expressed genes (DEGs) were identified by pairwise comparison of the analyzed samples using the following criteria: *P*-value ≤ 0.05 and |Log_2_foldchange(FC)|≥ 1. Using these criteria, the expression of differentially expressed genes (DEGs) were obtained, and the results were shown in Fig. 3. There were 3625 DEGs in X11 vs. X7 (1461 up-regulated and 2164 down-regulated), 1595 DEGs in X24 vs. X7 (841 up-regulated and 754 down-regulated), 2366 DEGs in X11 vs. X24 (724 up-regulated and 1642 down-regulated) at 8 DAH, 2616 DEGs in X11 vs. X7 (1317 up-regulated and 1299 down-regulated), 2756 DEGs in X24 vs. X7 (1320 up-regulated and 1436 down-regulated), 2608 DEGs in X11 vs. X24 (1399 up-regulated and 1209 down-regulated) at 12 DAH, 2994 DEGs in X11 vs. X7 (1368 up-regulated and 1626 down-regulated), 3830 DEGs in X24 vs. X7 (812 up-regulated and 3018 down-regulated) and 3509 DEGs in X11 vs. X24 (2535 up-regulated and 974 down-regulated) at 16 DAH.

Genes were differentially expressed in X11 vs. X7 and X24 vs. X7, but not in X11 vs. X24, these DEGs were only differentially expressed between high chalkiness and low chalkiness (DEG_HL_), and they might be related to chalkiness. Further analysis showed that there were 1020 DEG_HL_ at 8 DAH (491 up-regulated and 529 down-regulated), 1000 DEG_HL_ at 12 DAH (460 up-regulated and 540 down-regulated) and 1088 DEG_HL_ at 16 DAH (397 up-regulated and 691 down-regulated) (Supplementary Fig. 1A).

### Functional classification by Gene Ontology (GO) and Kyoto Encyclopedia of Genes and Genomes (KEGG) pathway mapping

**Fig. 4:**
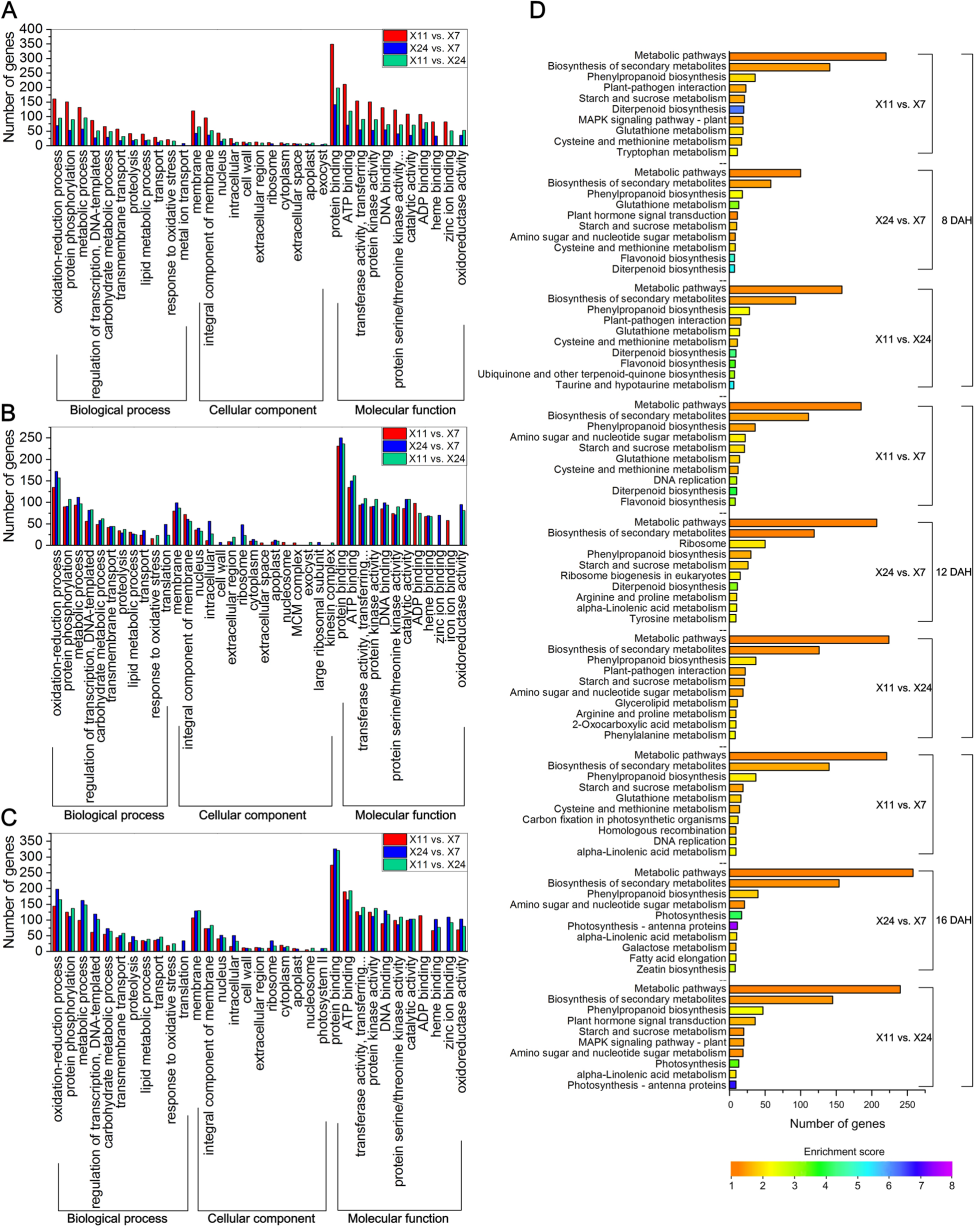
Comparison of Gene Ontology (GO) classifications of DEGs at (**A**) 8 DAH, (**B**) 12 DAH and (**C**) 16 DAH. (**D**) KEGG pathway assignments of DEGs at 8 DAH, 12 DAH and 16 DAH, the top 10 categories are shown. DEGs were differentially expressed with statistical significance (P-value ≤ 0.05 and |Log_2_foldchange(FC)|≥ 1)

In order to evaluate the potential functions of these DEGs in caryopsis, Gene Ontology (GO) analysis was performed. In X11 vs. X7, X24 vs. X7 and X11 vs. X24, there were 550 DEGs, 340 DEGs and 399 DEGs at 8 DAH, 495 DEGs, 588 DEGs and 510 DEGs at 12 DAH, 581 DEGs, 602 DEGs and 583 DEGs at 16 DAH, which were assigned to at least one term in ‘biological process’, ‘cellular component’ and ‘molecular function’ categories (Fig. 4A-C). The GO functional enrichment analysis of DEGs showed that the most enriched genes were ‘molecular function’, the second was ‘biological process’, and the least was ‘cellular component’. In the ‘biological process’ category, oxidation–reduction process (GO:0,055,114), protein phosphorylation (GO:0,006,468) and metabolic process (GO:0,008,152) were prominently represented at 8 DAH, 12 DAH and 16 DAH. In the ‘cellular component’ category, membrane (GO:0,016,020), integral component of membrane (GO:0,016,021) and nucleus (GO:0,005,634) were prominently represented at 8 DAH, 12 DAH and 16 DAH, while protein binding (GO:0,005,515), ATP binding (GO:0,005,524) and transferase activity, transferring phosphorus-containing groups (GO:0,016,772) dominated the ‘molecular function’ category at 8 DAH, 12 DAH and 16 DAH.

The GO functional enrichment analysis of DEG_HL_ showed that in the ‘biological’ process category, metabolic process (GO:0,008,152), oxidation–reduction process (GO:0,055,114), protein phosphorylation (GO:0,006,468), carbohydrate metabolic process (GO:0,005,975), regulation of transcription, DNA-templated (GO:0,006,355) and lipid metabolic process (GO:0,006,629) were prominently represented at 8 DAH, 12 DAH and 16 DAH. In the ‘cellular component’ category, integral component of membrane (GO:0,016,021), membrane (GO:0,016,020) and nucleus (GO:0,005,634) were prominently represented at 8 DAH, 12 DAH and 16 DAH, while protein binding (GO:0,005,515), ADP binding (GO:0,043,531), ATP binding (GO:0,005,524), transferase activity, transferring phosphorus-containing groups (GO:0,016,772) and protein kinase activity (GO:0,004,672) dominated the ‘molecular function’ category at 8 DAH, 12 DAH and 16 DAH (Supplementary Fig. 1B). The results indicated that enzyme activity and metabolic regulation affect chalkiness formation.

In order to identify metabolic pathways in which DEGs involved and enriched, pathway-based analysis was performed by using the KEGG pathway database. As shown in Fig. 4D, DEGs mainly belonged to the following KEGG pathways: metabolic pathways (path:dosa01100), biosynthesis of secondary metabolites (path:dosa01110), phenylpropanoid biosynthesis (path:dosa00940), starch and sucrose metabolism (path:dosa00500) and amino sugar and nucleotide sugar metabolism (path:dosa00520).

The KEGG pathway assignments analysis of DEG_HL_ showed that DEG_HL_ mainly belonged to metabolic pathways (path:dosa01100), biosynthesis of secondary metabolites (path:dosa01110) at 8 DAH, and mainly belonged to metabolic pathways (path:dosa01100), biosynthesis of secondary metabolites (path:dosa01110), phenylpropanoid biosynthesis (path:dosa00940) and starch and sucrose metabolism (path:dosa00500) at 12 DAH and 16 DAH (Supplementary Fig. 1C). The metabolic pathways exhibited the most DEG_HL_, suggesting that metabolic pathways play significant roles in growth and development of rice grain. The second largest number of DEG_HL_ related to the biosynthesis of secondary metabolites, indicating that biosynthesis of secondary metabolites is also important for rice grain, because the growth and development of rice grain are also based on the accumulation of starch and protein. Starch and sucrose metabolism were represented in large numbers at 12 DAH and 16 DAH. That was unsurprising because metabolism of sugar plays an important role in the growth and development of rice, hence starch and sucrose are tightly related to chalkiness formation. At 8 DAH and 12 DAH, the number of up-regulated DEG_HL_ in KEGG pathway was more than that of down-regulated DEG_HL_, but at 16 DAH, the number of up-regulated DEG_HL_ was significantly less than that of down-regulated DEG_HL_.

### Gene expression profiles in starch/sucrose/protein metabolism during chalkiness formation

**Fig. 5:**
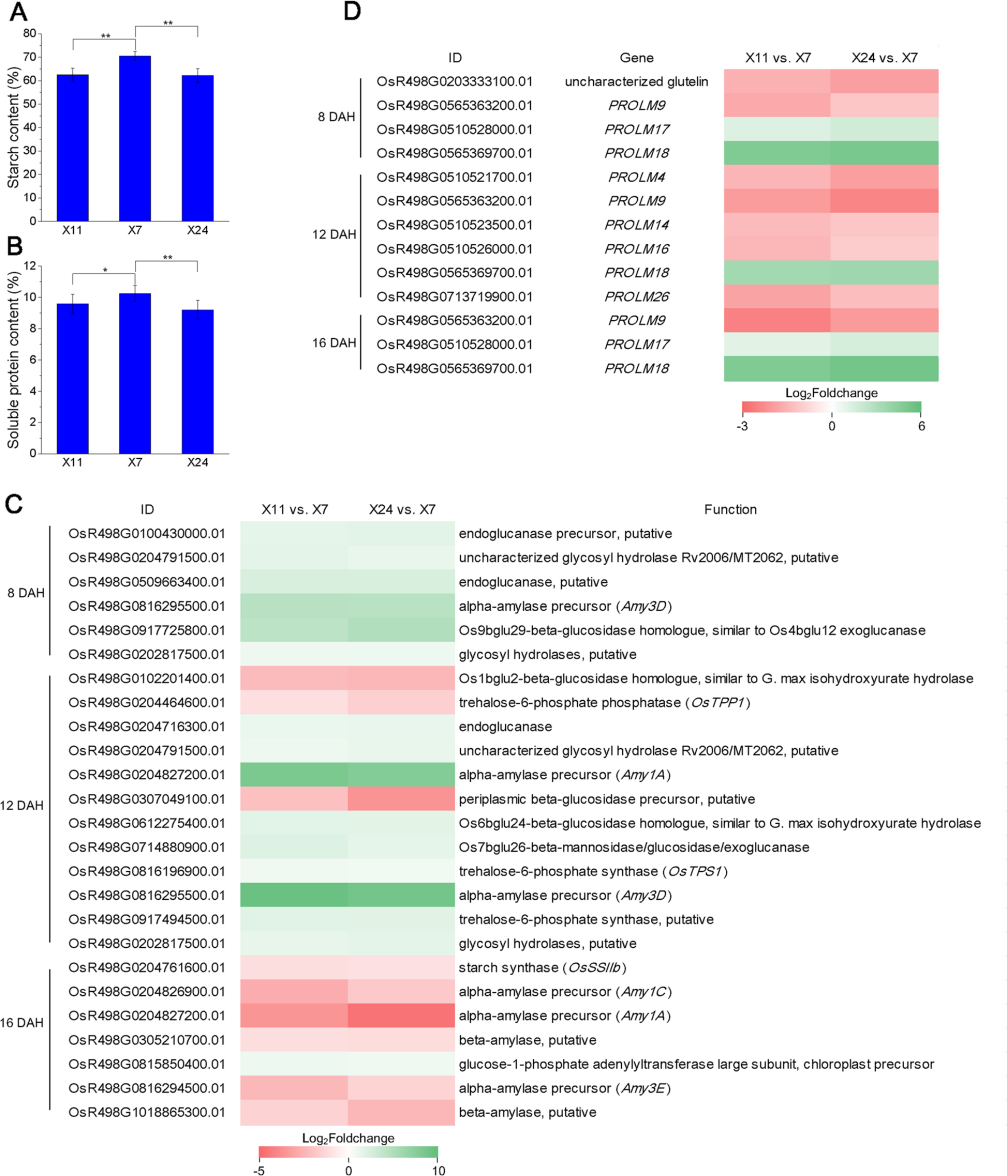
**A** The starch content in of mature grain in X11, X7 and X24. **B** The starch and soluble protein content in of mature grain in X11, X7 and X24, data shown as means ± SD of three biological replicates, asterisks indicate a significant difference based on a Dunnett’s test. * significant difference at 5% level (*P* < 0.05); ** significant difference at 1% level (*P* < 0.01). **C** DEG_HL_ involved in starch and sucrose metabolism at 8 DAH, 12 DAH and 16 DAH, which are shown as log_2_Foldchange levels. DEG_HL_ were differentially expressed with statistical significance (*P*-value ≤ 0.05 and |Log_2_foldchange(FC)|≥ 1)

After flowering, starch, protein and lipid begin to fill caryopsis. In order to analyze the difference in substance content between high and low chalkiness, we measured the starch and soluble protein content in mature grains. The starch and soluble protein content in X7 mature grain was higher than that in X11 and X24. The total starch content in X7 was 70.53%, which was higher than that of 62.53% in X11 and 62.15% in X24. The soluble protein content in X7 was 10.24%, which was higher than that of 9.58% and 9.21% in X11 and X24 (Fig. 5A-B). The results showed that the decrease of starch and soluble protein contents is one of the reasons for chalkiness formation.

Since grains of X11 and X24 contained lower starch content compared with X7, we speculated that genes involved in starch and sucrose metabolism might be differentially expressed between high and low chalkiness caryopsis. The transcriptome analysis found that there were 6 DEG_HL_ at 8 DAH, 12 DEG_HL_ at 12 DAH and 7 DEG_HL_ at 16 DAH involved in starch and sucrose metabolism (Fig. 5C). In these DEG_HL_, at 8 DAH, alpha-amylase gene *Amy3D*, 2 glycosyl hydrolase genes, 2 endoglucanase genes and 1 beta-glucosidase homologue gene were up-regulated. At 12 DAH, 2 alpha-amylase gens *Amy1A* and *Amy3D* were significant up-regulated, 1 beta-glucosidase homologue gene and 1 beta-glucosidase gene were up-regulated. Other 2 beta-glucosidase homologue gene were down-regulated, and 2 glycosyl hydrolase genes were up-regulated. Alpha-amylase and glycosyl hydrolase are the key enzymes in the hydrolysis of starch, endoglucanase is the main component of cellulase system, and beta-glucosidase promotes the degradation of cellulose. Their differential expressions suggested that starch degradation and cellulose metabolic are associated with chalkiness formation at the early and middle stages of grain filling. In addition, 3 key enzyme genes in trehalose synthesis were differentially expressed at 12 DAH, indicating that the trehalose metabolism is also involved in chalkiness formation. At 16 DAH, alpha-amylase genes *Amy1C*, *Amy1A*, *Amy3E* and 2 beta-amylase genes were down-regulated, and starch synthase gene *OsSSIIb* was also down-regulated. Thus we speculated that starch synthesis and hydrolysis decrease at the late stage of grain filling in high chalkiness caryopsis. The results showed that different genes in starch and sugar metabolism are differentially expressed at different stages of grain filling, and these dynamic regulatory processes eventually result in chalkiness formation.

In addition, protein accumulating between starch granules is the second abundant component in rice endosperm [48]. Increasing evidences suggested the importance of proteins in chalkiness formation. A large amount of storage proteins, such as glutelins, prolamins and α-globulin, were found to be accumulated in mature rice seeds [41]. In our results, soluble protein contents in X24 and X11 were lower than that in X7. We speculated that protein metabolism is also an important process for chalkiness formation. Among seed-specific storage proteins, there were 4 DEG_HL_ at 8 DAH, 6 DEG_HL_ at 12 DAH and 3 DEG_HL_ at 16 DAH (Fig. 5D). In these DEG_HL_, only one glutelin gene was down-regulated at 8 DAH, and no differentially expressed glutenin genes were found at 12 DAH and 16 DAH. In addition, the expression of *PROLM17* was up-regulated at 8 DAH and 16 DAH, and *PROLM18* was up-regulated at 8 DAH, 12 DAH and 16 DAH, while the other prolamin genes were down-regulated at 8 DAH, 12 DAH and 16 DAH. These results showed that prolamin and glutenin associated with the storage proteins might be related to chalkiness formation.

### Phytohormonal contents and related gene expression differences during chalkiness formation

**Fig. 6:**
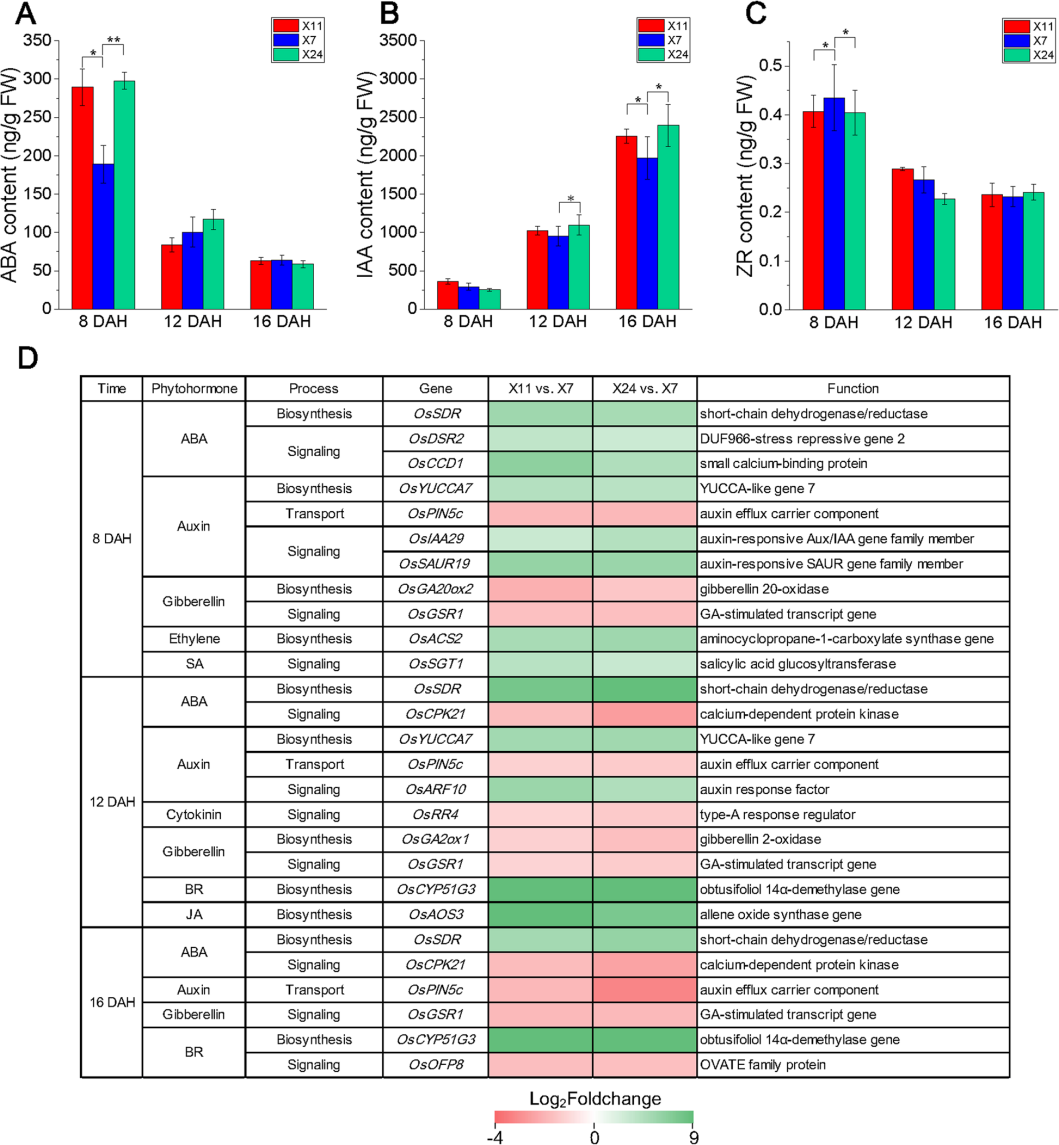
The phytohormonal contents (**A**) ABA, (**B**) IAA and (**C**) ZR at 8 DAH, 12 DAH and 16 DAH, data shown as means ± SD of three biological replicates, asterisks indicate a significant difference based on a Dunnett’s test. * significant difference at 5% level (*P* < 0.05); ** significant difference at 1% level (*P* < 0.01). (**D**) Expression patterns of phytohormone-related DEG_HL_ at 8 DAH, 12 DAH and 16 DAH, which are shown as log_2_Foldchange levels. DEG_HL_ were differentially expressed with statistical significance (P-value ≤ 0.05 and |Log_2_foldchange(FC)|≥ 1)

It is well known that phytohormones act as signaling molecules in plants growth and development [49, 50]. We thus measured ABA, IAA and ZR contents (Fig. 6A-C) in caryopsis at 8 DAH, 12 DAH and 16 DAH, respectively. The overall trend of ABA content in caryopsis was 8 DAH > 12 DAH > 16 DAH. ABA content in X11 and X24 were 289.34 ng/g and 297.71 ng/g, which were lower than that of 188.88 ng/g in X7 at 8 DAH. There was no significant difference in the ABA content between high and low chalkiness caryopsis at 12 DAH and 16 DAH. The trend of IAA content in caryopsis was 8 DAH < 12 DAH < 16 DAH. IAA contents in X11 and X24 were 1023.74 ng/g and 1097.86 ng/g, which were higher than that of 954.69 ng/g in X7 at 12 DAH. At 16 DAH, IAA contents in X11 and X24 were 2254.95 ng/g and 2395.91 ng/g, which were higher than that of 1969.66 ng/g in X7. There was no significant difference in the IAA content between high and low chalkiness caryopsis at 8 DAH. The trend of cytokine zeatin riboside (ZR) content in caryopsis was 8 DAH > 12 DAH > 16 DAH, and there was no significant difference.

In order to analyze the regulatory function of phytohormones in chalkiness formation, we analyzed the expression patterns of genes related to phytohormonal biosynthesis and signaling (Fig. 6D). It was found that many DEG_HL_ are related to phytohormones in rice (Fig. 6D). *OsSDR*, a gene related to ABA biosynthesis was up-regulated at three sampling dates. ABA signaling-related genes *OsDSR2* and *OsCCD1* were up-regulated at 8 DAH. *OsCPK21* was down-regulated at 12 DAH and 16 DAH. The differential gene expression led to the significant change of ABA content, which showed a decrease tendency from 8 to 16 DAH (Fig. 6A). This suggested that ABA exert an important regulatory function during caryopsis development, especially at the early grain filling stage. An auxin biosynthesis gene *OsYUCCA7* was up-regulated at 8 DAH and 12 DAH. Auxin responsive genes *OsIAA29* and *OsSAUR19* were up-regulated at 12 DAH. Auxin response factor (ARF) gene *OsARF10* was up-regulated at 12 DAH. Auxin transport gene *OsPIN5c* was down-regulated at three sampling dates. These results indicated the regulation function of auxin during caryopsis development, especially at the late grain filling stage. Among gibberellin-related DEG_HL_, a positive regulator gene *OsGSR1* was down-regulated at three sampling dates. Rate-limiting enzyme gene of gibberellin biosynthesis, *OsGA20ox2* was down-regulated at 8 DAH, and *OsGA2ox1* were down-regulated at 12 DAH. Meanwhile, the cytokinin-related genes showed relatively little difference in caryopsis between high and low chalkiness at the early and late grain filling stages, suggesting that cytokinin might function in the middle period at grain filling stage. In addition, BR biosynthesis related gene *OsCYP51G3* was up-regulated at 12 DAH and 16 DAH, and a positive regulator gene in BR signaling *OsOFP8* was down-regulated. We speculated that BR mainly plays the regulatory role in chalkiness formation at the middle and late stages of grain filling. Ethylene (ETH) biosynthesis related gene *OsACS2* and salicylic acid (SA) signaling related gene *OsSGT1* were up-regulated at 8 DAH, and jasmonates (JA) biosynthesis related gene *OsAOS3* was up-regulated at 12 DAH, indicating that these three phytohormones might play roles at the early and middle stages of grain filling. The differences in phytohormonal levels and distinct patterns of phytohormone-related gene expression at 8 DAH, 12 DAH and 16 DAH revealed complex and distinct roles during caryopsis development. Obviously, phytohormones are the important regulators in chalkiness formation, and thus the underlying regulation mechanism needs to be further studied.

### Differential gene expression of transcription factors interacted with phytohormones to regulate chalkiness formation

Generally, phytohormones regulate the plant growth and development, and mediate the responses to the environment through interaction with multiple regulators. Among them, the transcription factors (TFs) have become the focus. In order to screen phytohormones-interacted TFs involved in chalkiness formation, the expression profile of TFs was analyzed in detail. The results showed that 73 *MYB*, 8 GRAS, 16 *bZIP*, 52 *AP2/EREBP*, 7 *GATA*, 2 *NAC*, 5 *MAD*S, 49 *WRKY* and 9 *TCP* were differentially expressed in at least one group of pairwise comparison (Table 1).

**Table 1:** DEGs encoding TFs differentially expressed in X11 vs. X7, X24 vs. X7, and X11 vs. X24 at 8 DAH, 12 DAH and 16 DAH

Family	8 DAH						12 DAH						16 DAH					
	X11 vs. X7		X24 vs. X7		X11 vs. X24		X11 vs. X7		X24 vs. X7		X11 vs. X24		X11 vs. X7		X24 vs. X7		X11 vs. X24	
	Up	Down	Up	Down	Up	Down	Up	Down	Up	Down	Up	Down	Up	Down	Up	Down	Up	Down
*MYB*	14	6	9	1	4	7	6	2	15	7	10	13	12	5	2	17	18	3
*GRAS*	3	1	0	0	2	0	1	0	2	1	1	1	0	0	0	8	7	0
*bZIP*	2	3	0	0	0	2	2	3	0	1	3	1	0	2	0	3	4	0
*AP2*	8	8	5	2	2	10	5	3	4	5	8	4	1	6	0	26	19	1
*GATA*	0	0	0	0	0	0	0	0	0	2	1	0	0	1	0	6	4	0
*NAC*	0	0	0	0	0	0	0	0	2	0	0	2	0	0	0	0	0	0
*MADS*	1	1	0	0	1	0	0	1	0	1	0	1	2	0	0	0	0	1
*WRKY*	10	6	3	2	6	3	7	4	8	8	8	11	6	6	0	19	17	3
*TCP*	0	0	0	0	0	1	0	0	1	0	0	0	0	0	0	9	6	0

Numbers of DEGs encoding TFs differentially expressed with statistical significance (*P*-value ≤ 0.05 and |Log_2_foldchange(FC)|≥ 1) are shown; Up: up-regulated; Down: down-regulated

**Fig. 7:**
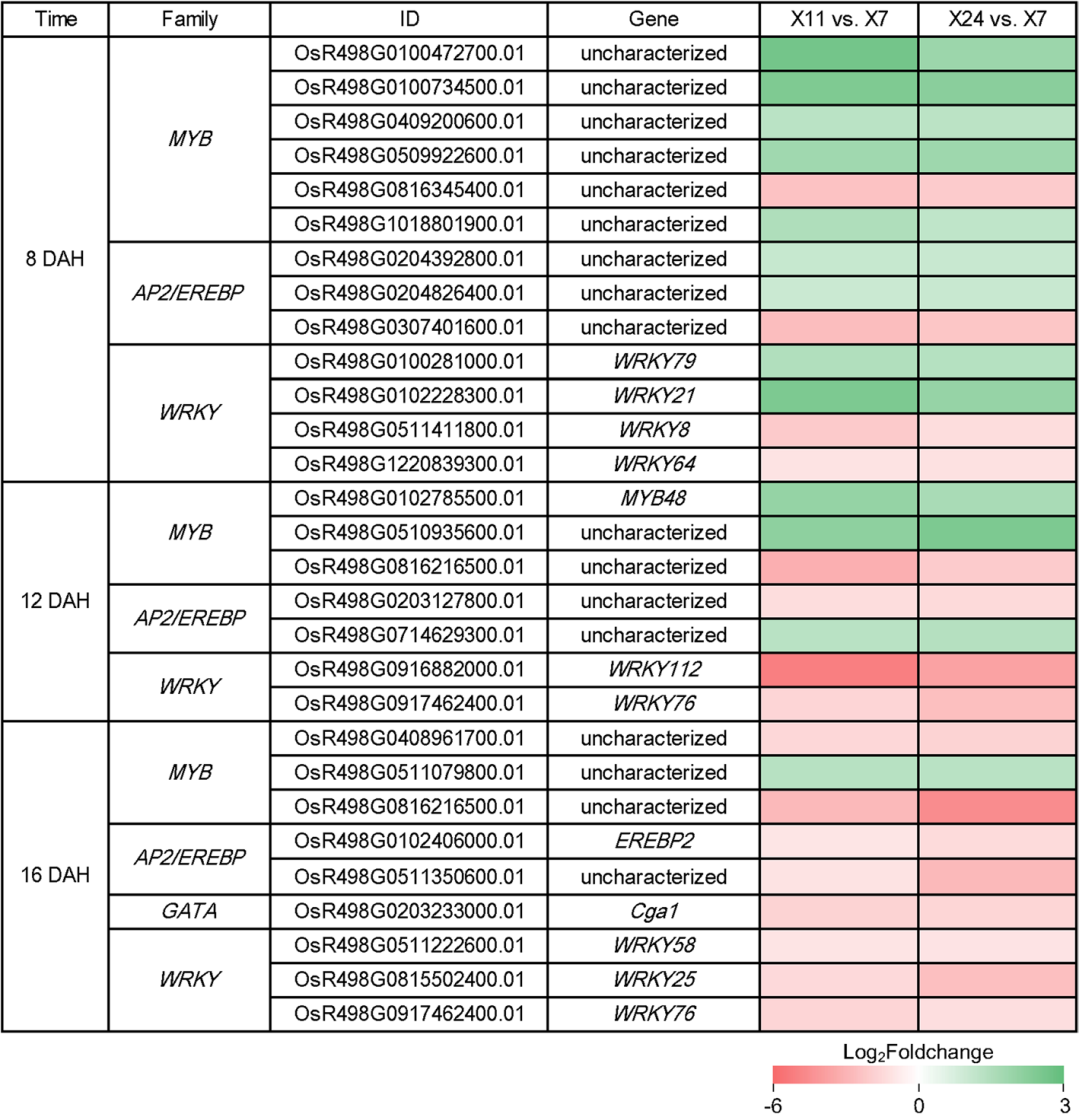
Expression patterns of DEG_HL_ encoding TFs obtained at 8 DAH, 12 DAH and 16 DAH, which are shown as log_2_Foldchange levels. DEG_HL_ were differentially expressed with statistical significance (P-value ≤ 0.05 and |Log_2_foldchange(FC)|≥ 1)

Further analysis found that 13 DEG_HL_ at 8 DAH, 7 DEG_HL_ at 12 DAH and 9 DEG_HL_ at 16 DAH were differentially expressed with distinct patterns (Fig. 7), implying their functions at the specific stages of grain filling. 5 members of *MYB* were up-regulated but 1 member was down-regulated at 8 DAH; 2 members of *MYB* were up-regulated but 1 member was down-regulated at 12 DAH; 1 member of *MYB* was up-regulated but 2 members were down-regulated at 16 DAH. MYB family were reported as important regulators in plant tolerance to abiotic stresses and development [51]. *OsMYB48,* which was reported to play a positive role in drought and salinity tolerance by regulating stress-induced ABA synthesis [52], was up-regulated at 12 DAH, suggesting its potential role in the middle period of grain filling stage. 2 *AP2/EREBP* members were up-regulated and 1 member was down-regulated at 8 DAH; 1 *AP2/EREBP* member was up-regulated and 1 member down-regulated at 12 DAH; 2 *AP2/EREBP* members were down-regulated at 16 DAH. *OsEREBP2*, which is significantly inducible in response to ABA [53], was down-regulated at 16 DAH, indicating that *OsEREBP2* might play an important role in the transcriptional network and contribute to the development of caryopsis. An AP2/EREBP member RSR1 was reported to be involved in rice chalkiness formation by regulating starch synthesis [35]. It suggested that the differentially expression of the *AP2/EREBP* might contribute to the development of caryopsis. *WRKYs* showed dramatic changes from 8 to 16 DAH. *WRKY102* and *WRKY21* were up-regulated, and *WRKY8* and *WRKY64* were down-regulated at 8 DAH. *WRKY116* and *WRKY72* were down-regulated at 12 DAH, and *WRKY58*, *WRKY25* and *WRKY76* were down-regulated at 16 DAH. A number of studies have shown that the majority of WRKY family members are involved in response to biotic and/or abiotic stresses [54–57]. *OsWRKY8*, which was reported to be induced by ABA [58], was down-regulated at 8 DAH. *OsWRKY76* [59], which was reported to be induced by ABA, was down-regulated at 12 DAH and 16 DAH. The results indicated that OsWRKY8 and OsWRKY76 might play regulating function at the whole grain filling stage through the interaction with ABA. *Cga1* (*GATA* member) was reported to be induced the expression by gibberellin [60], it showed decreased expression at 16 DAH. These results suggested that TFs show distinct patterns at different stages to regulate the development of caryopsis and chalkiness formation through the interactions with phytohormones.

### Differential gene expression of kinases interacted with phytohormones to regulate chalkiness formation

Some protein kinases (PKs) are involved in phytohormonal signaling [61]. Plant AGC kinases mediate auxin signaling to regulate growth and morphogenesis of plant [62]. TKL were reported to play crucial roles in plant response to various external signals [63]. For example, the leucine-rich repeat (LRR) receptor-like PKs BRI1 and BAK1 were involved in BR signal transduction in *Arabidopsis* [64, 65]. In addition, OsRPK1 participated in ABA signaling pathway [66]. Several CDPKs had been shown to be essential factors in abiotic stress tolerance by modulating ABA signaling and reducing the accumulation of reactive oxygen species [67]. OsWNK9 plays a role in ABA-mediated stress tolerance mechanisms [68]. The GO functional enrichment analysis of DEGs showed that protein phosphorylation and protein serine/threonine kinase activity were high enriched at 8 DAH, 12 DAH and 16 DAH (Fig. 4A-C). Further analysis showed that 5 *AGC*, 32 *CAMK*, 5 *CGMC*, 2 *CK1*, 17 *STE* and 30 *TKL* were differentially expressed in at least one group of pairwise comparison (Table 2).

**Table 2:** DEGs encoding PKs differentially expressed in X11 vs. X7, X24 vs. X7, and X11 vs. X24 at 8 DAH, 12 DAH and 16 DAH

Family	8 DAH						12 DAH						16 DAH					
	X11 vs. X7		X24 vs. X7		X11 vs. X24		X11 vs. X7		X24 vs. X7		X11 vs. X24		X11 vs. X7		X24 vs. X7		X11 vs. X24	
	Up	Down	Up	Down	Up	Down	Up	Down	Up	Down	Up	Down	Up	Down	Up	Down	Up	Down
*AGC*	0	1	0	1	0	0	1	0	1	1	0	0	0	0	2	2	1	2
*CAMK*	5	6	3	3	3	5	2	4	0	5	6	1	3	4	2	7	6	4
*CGMC*	0	3	0	0	0	1	0	2	0	4	1	0	1	0	0	0	0	1
*CK1*	0	0	0	0	0	0	0	1	0	1	0	0	0	0	1	0	0	0
*FGGY*	0	0	0	1	0	0	0	0	0	1	0	0	0	0	0	0	0	0
*SET*	1	5	1	0	3	1	1	1	2	2	2	9	2	3	1	5	5	0
*TKL*	8	2	2	3	5	1	3	2	5	2	4	2	9	3	0	6	11	3

Numbers of DEGs encoding PKs differentially expressed with statistical significance (*P*-value ≤ 0.05 and |Log_2_foldchange(FC)|≥ 1) are shown; Up: up-regulated; Down: down-regulated

**Fig. 8:**
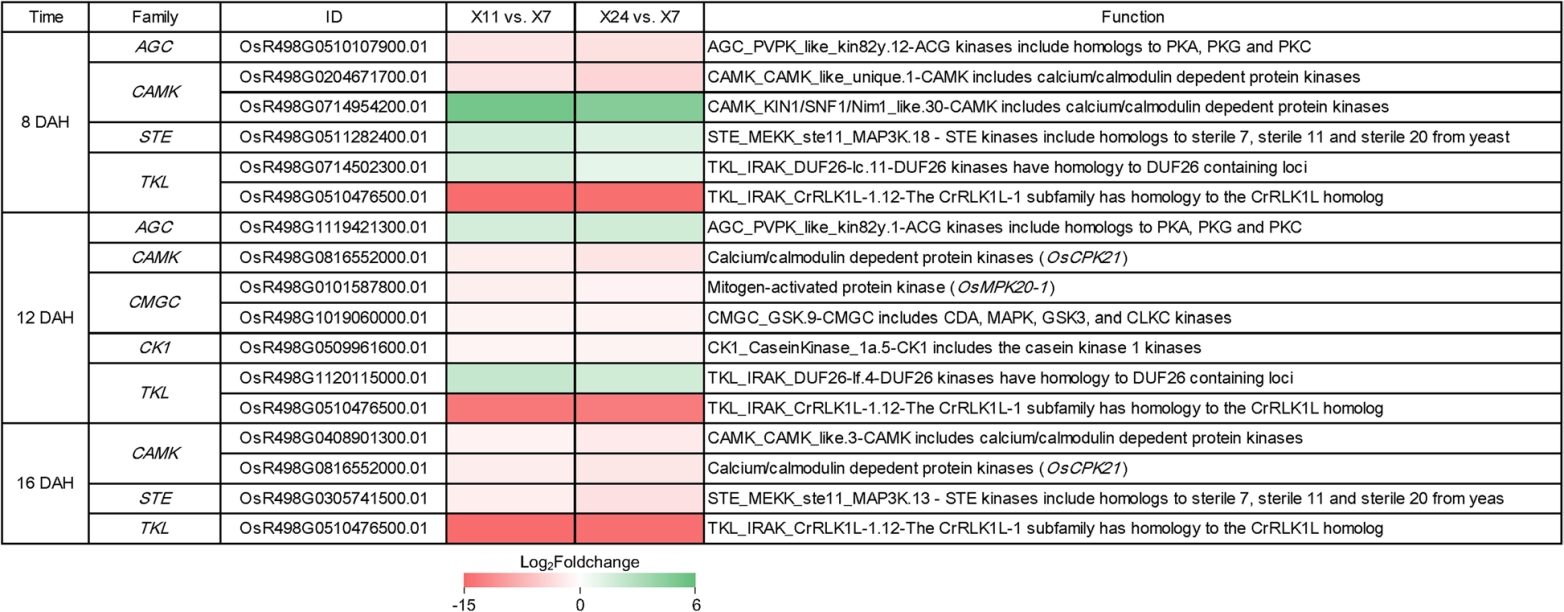
Expression patterns of DEG_HL_ encoding PKs obtained at 8 DAH, 12 DAH and 16 DAH, which are shown as log_2_Foldchange levels. DEG_HL_ were differentially expressed with statistical significance (*P*-value ≤ 0.05 and |Log_2_foldchange(FC)|≥ 1)

In these PKs, there were 6 DEG_HL_ at 8 DAH, 7 DEG_HL_ at 12 DAH and 4 DEG_HL_ at 16 DAH (Fig. 8). At 8 DAH, 1 *CAMK*, 1 *STE* and 1 *TKL* were up-regulated, 1 *AGC*, 1 *CAMK* and 1 *TKL* were down-regulated. At 12 DAH, 1 *AGC* and 1 *TKL* were up-regulated, 2 *CAMK*, 2 *CGMC*, 1 *CK1* and 1 *TKL* were down-regulated. At 16 DAH, 2 *CAMK*, 1 *STE* and 1 *TKL* were down-regulated. The results showed that the differential expression of *CAMK* and *TKL* were found at 8 DAH, 12 DAH and 16 DAH, and the number of DEG_HL_ in these two groups were more than other groups. Meanwhile, *TKL* member (*OsR498G0510476500.01*) was significantly down-regulated (Log_2_Foldchange < -10) at three sampling dates. *OsCPK21*, which was involved in the response to ABA [69], was down-regulated at 12 DAH and 16 DAH. Therefore, we speculated that some PKs interacted with phytohormones might be important factors in the regulation of the caryopsis development and chalkiness formation.

### Validation of DEG_HL_ by real-time PCR analysis

**Fig. 9:**
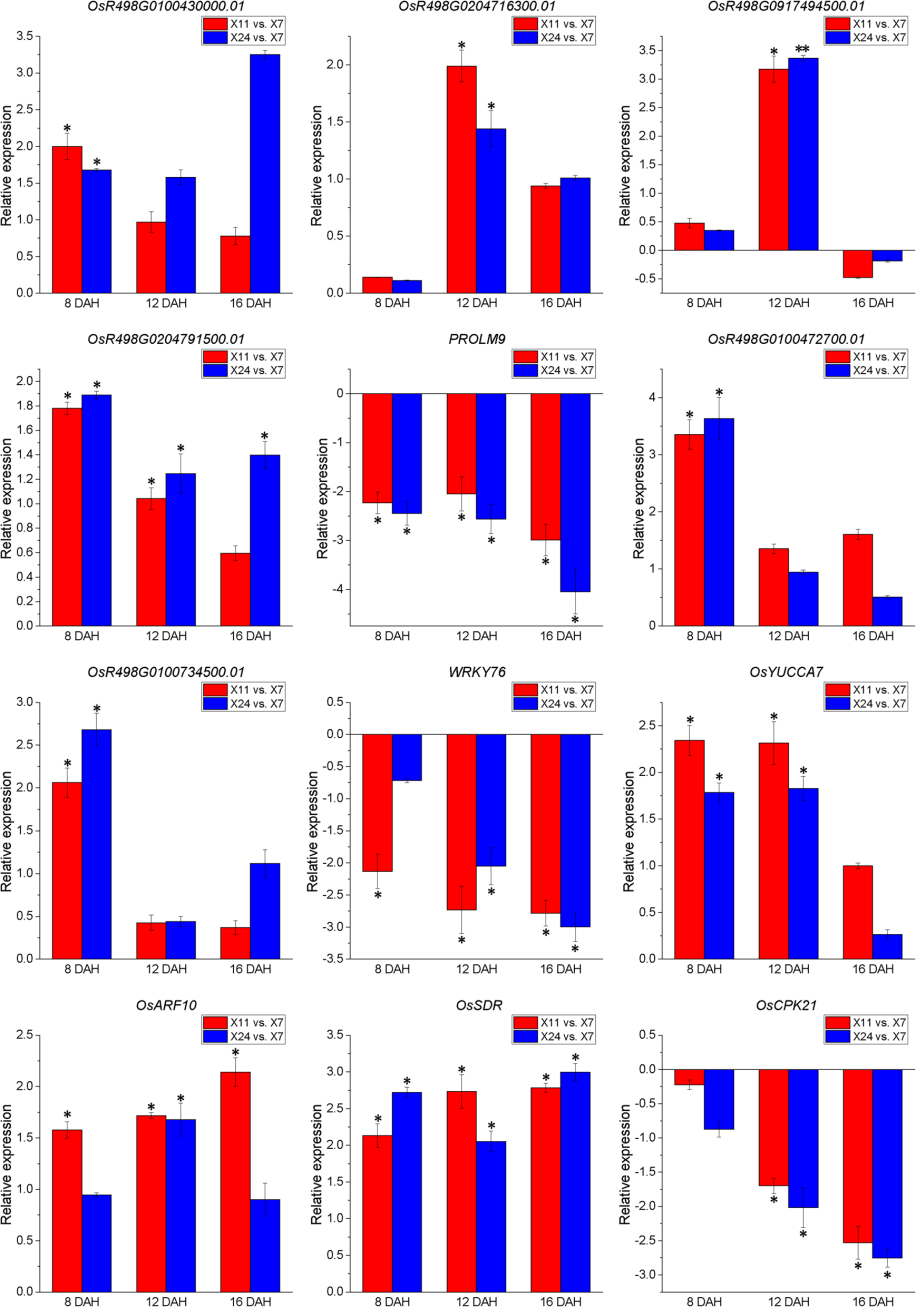
Expression patterns of 12 DEG_HL_ confirmed by real-time PCR, which are shown as log_2_Foldchange levels. DEG_HL_ were differentially expressed with statistical significance (*P*-value ≤ 0.05 and |Log_2_foldchange(FC)|≥ 1). Data shown as means ± SD of three biological replicates. Asterisks indicate a significant difference based on a Dunnett’s test. * significant difference at 5% level (*P* < 0.05); ** significant difference at 1% level (*P* < 0.01)

In order to validate the RNA-seq data, 12 DEG_HL_ were selected to confirm expression by real-time PCR analysis, including 5 DEG_HL_ involved in starch/sucrose and protein metabolism (*OsR498G0100430000.01*, *OsR498G0204716300.01*, *OsR498G0917494500.01*, *OsR498G0204791500.01* and *PROLM9*), 3 DEG_HL_ encoding TFs (*OsR498G0100472700.01*, *OsR498G0100734500.01* and *WRKY76*), 3 DEG_HL_ related to phytohormonal biosynthesis and signaling (*OsYUCCA7*, *OsARF10* and *OsSDR*), 1 DEG_HL_ encoding PKs (*OsCPK21*). For these 12 DEG_HL_, the expression patterns in real-time PCR results were similar to RNA-Seq data (Fig. 9), and the results also validate the reliability of RNA-Seq data. In future studies, we will focus on these DEG_HL_ to investigate their regulation of chalkiness formation.

## Discussion

### Chalkiness formation is a dynamic process

Chalkiness is closely related to the grain filling dynamics, and disordered dynamics of grain filling is an important reason for chalkiness formation. In our research, the color of glumes changed from green to yellow at 16 DAH until mature, caryopsides were gradually hardened since 16 DAH. Since 20 DAH, the caryopsis of X7 began to translucent, while the chalkiness in X11 and X24 began to form at the belly of caryopsis (Fig. 2A-B). It indicated that chalkiness formation is a dynamic process during the grain filling of caryopsis. The endosperms of X11 and X24 carried small round shaped and loosely packed starch granules with air spaces, while endosperms of X7 were filled with large and tightly packed starch granules at 8 DAH (Fig. 2C). The endosperms at 12 DAH, 16 DAH, 20 DAH and 24 DAH were similar to that at 8 DAH. It indicated that the different size, shape and arrangement of starch granules between high and low chalkiness caryopsis at the early grain filling stage, result in the contrasted chalkiness formation. There were 1020 DEG_HL_ at 8 DAH, 1000 DEG_HL_ at 12 DAH and 1088 DEG_HL_ at 16 DAH (Supplementary Fig. 1A-D). The KEGG pathway analysis of DEG_HL_ showed that DEG_HL_ mainly belong to metabolic pathways, biosynthesis of secondary metabolites at 8 DAH, and DEG_HL_ mainly belong to metabolic pathways, biosynthesis of secondary metabolites, phenylpropanoid biosynthesis, starch and sucrose metabolism at 12 DAH and 16 DAH (Supplementary Fig. 1F). These DEG_HL_ were closely related to caryopsis development, suggesting that chalkiness formation is a dynamic process regulated by multiple genes.

### Starch/sucrose/protein metabolism is involved in chalkiness formation

Chalkiness formation is controlled by multiple complex regulators. Starch is the end-product of photosynthesis and is stored as energy reserves in rice grain. Amylose and amylopectin are two main components of starch [70]. Amylose is synthesized by AGPase and GBSS, and amylopectin is synthesized by AGPase, SS, BE and DBE [71, 72]. Mutations of *SSIIIa* [23] and *BEIIb* [23, 24] involved in the starch biosynthesis pathway showed defective amyloplast development and chalky phenotype. Sucrose is also important for caryopsis development as sources of energy and carbon skeletons for cell wall and starch biosynthesis [73, 74]. UGPase1 regulates the utilization of sucrose, malfunction of *UGPase1* gene causes the decrease of amylopectin long chain and the change of starch structure, resulting in chalkiness formation [75]. In addition, other factors related to the development of amyloplast have also been reported, such as *FLO*2 [27], *FLO6* [28], *FLO7* [76], *FLO10* [77], *FLO16* [78], *SSG4* [79] and *SSG6* [80], *OsAGPL2* [25], *OsPho1* [29], *ISA1* [30]. In our results, alpha-amylase genes were up-regulated at the early (*Amy3D*) and middle stages (*Amy1A* and *Amy3D*) of grain filling, but down-regulated at the late stage (*Amy1C*, *Amy1A* and *Amy3E*) of grain filling in high chalkiness grains. Starch synthesis genes (*OsSSIIb*) were down-regulated at the late grain filling stage. These results suggested that starch and sucrose metabolism are closely related to chalkiness formation. Meanwhile, glycosyl hydrolase, the key enzyme in the hydrolysis of starch, were up-regulated at 8 DAH (*OsR498G0204791500.01* and *OsR498G0202817500.01*) and 12 DAH (*OsR498G0204791500.01* and *OsR498G0202817500.01*) (Fig. 5C). Two beta-amylase genes (*OsR498G0305210700.01* and *OsR498G1018865300.01*) were down-regulated at 16 DAH. The results suggested that starch was degraded by alpha-amylase and glycosyl hydrolase at the early and middle grain filling stages, starch synthesis and hydrolysis decreased at the late grain filling stage in high chalkiness caryopsis. Moreover, endoglucanase and glucosidase related to degradation of cellulose were differentially expressed at 8 DAH (*OsR498G0100430000.01*, *OsR498G0509663400.01* and *OsR498G0917725800.01*) and 12 DAH (*OsR498G0102201400.01*, *OsR498G0204716300.01*, *OsR498G0307049100.01*, *OsR498G0612275400.01* and *OsR498G0714880900.01*), and trehalose synthase genes (*OsR498G0204464600.01*, *OsR498G0816196900.01* and *OsR498G0917494500.01*) were also differentially expressed at 12 DAH (Fig. 5C), indicating that non-starch polysaccharide metabolism might also be involved in chalkiness formation.

The abnormal expression of protein metabolism genes, such as *PDIL1-1* [38], *BiP1* [39, 40], *OsVPS9A* [41] and *OsRAB5A* [42], can cause chalkiness. Overexpression of *Chalk5* affects the endomembrane protein trafficking system, resulting in an abnormal decrease in protein body number, and causing air spaces among starch granules and protein bodies [10]. Two genes encoding 13 kDa prolamin were reported to be down-regulated in the chalky grains [3, 18, 32], and lower concentration of prolamins and globulins in the chalky part of grains were also reported in previous study [31]. In this study, *PROLM9* was down-regulated in chalkiness caryopsis at 8 DAH, 12 DAH and 16 DAH; *PROLM4*, *PROLM14*, *PROLM16* and *PROLM26* were down-regulated in chalkiness caryopsis at 12 DAH (Fig. 5B). These results suggested that prolamin may also play a role in chalkiness formation.

### Phytohormones regulate the formation of chalkiness through a complex interactive network

The fact that complex processes of chalkiness formation are affected by multiple environmental factors implies a complex regulatory network mediating these processes in rice. Previous research found that the phytohormonal dynamics during rice endosperm development plays important roles in the grains quality. Among them, auxin and BRs are important for endosperm development [81–85]. Recently, a study showed that the increases of auxin, CKs and GAs levels lead to higher chalkiness, while BRs reduce chalkiness [44]. In this study, we found that ABA content was higher at 8 DAH and IAA content was higher at 16 DAH in high chalkiness caryopsis (Fig. 6A-B). The expression of some important genes in biosynthesis and signaling of ABA, auxin, GAs, ETH, SA, JA and BRs showed significant differences between high and low chalkiness caryopsis (Fig. 6D), particularly *OsSDR* (ABA biosynthesis), *OsYUCCA7* (auxin biosynthesis), *OsCYP51G3* (BR biosynthesis), *OsAOS*3 (JA biosynthesis), *OsDSR2* (ABA signaling), *OsCCD1* (ABA signaling), *OsIAA29* (auxin signaling), *OsSAUR19* (auxin signaling) and *OsARF10* (auxin signaling). Differential expression of genes in the phytohormonal signaling also results in the changes of responsive gene expression, which might be an additional reason for chalkiness formation.

TFs are very important in plant growth and development and many TFs function through the interaction with phytohormones. NF-YB1 regulate grain filling and endosperm development by interacting with ERF transcription factors and directly binding to three gene expression regulatory regions of sucrose transporter SUT1, SUT3 and SUT4 [33, 34]. Meanwhile, NF-YB1 binds directly and specifically to the G-box of the *Wx* promoter [86], and G-box is involved in the response to phytohormones [87]. Thus, phytohormones should also be involved in regulatory networks of NF-YB1 for grain filling and endosperm development. RSR1, an AP2/EREBP transcription factor, was found to negatively regulate the expression of type I starch synthesis genes (in seeds; sink tissues), among type I coexpressed genes, some of them are also involved in the response to GAs and ABA stimuli [35]. OsbZIP58 regulates starch biosynthesis in rice endosperm [36], and the interaction between OsLOL1 and OsbZIP58 influences GAs biosynthesis through the activation of *OsKO2 *via OsbZIP58 [88]. Interestingly, mutations of *NF-YB1* [33, 34], *NF-YC12* [89], *RSR1* [35], *OsbZIP58* [36] resulted in chalky phenotype. In our test, several genes encoding TFs were differentially expressed between high and low chalkiness caryopsis, including 6 *MYB*, 3 *AP2*, 4 *WRKY* at 8 DAH, 3 *MYB*, 2 *AP2* and 2 *WRKY* at 12 DAH, 3 *MYB*, 2 *AP2*, 1 *GATA* and 3 *WRKY* at 16 DAH (Fig. 7). In these TFs, OsMYB48 [52], OsEREBP2 [53], OsWRKY8 [58], OsWRKY76 [59] were reported to be involved in synthesis or signaling pathways of ABA. Therefore, we predicted these TFs might regulate the grain development and chalkiness formation by interacting with phytohormones.

PKs are major regulatory components in almost all cellular processes in eukaryotic cells [90]. Several PKs are involved in phytohormonal signaling transduction. OsCDPK1 plays key roles in negatively controlling the grain size, amylose content, and endosperm appearance [37]. Meanwhile, OsCDPK1 inhibits the feedback of GAs biosynthesis through down-regulating *GA3ox2* and *GA20ox1* [91]. Our results showed that several genes encoding PKs were differentially expressed between high and low chalkiness caryopsis (Fig. 8). Among these PKs, OsCPK21 was reported to be involved in the response to ABA [69], and thus it might influence the development of caryopsis by participating in the ABA signaling pathway. Plant AGC members are involved in auxin signaling [62], some TKL [66] and CAMK [67, 68] members are involved in ABA signaling. Therefore, the PKs may be involved in regulating the development of caryopsis and chalkiness formation by interacting with phytohormones.

### The interaction between different phytohormones regulate the formation of chalkiness

The reports showed that gradients of phytohormones are generated in the different seed compartments, and their ratios comprise the signals that induce/inhibit particular processes in seed development [92]. ABA can improve ADP-glucose pyrophosphorylase and amylase (SBE) activity in grain, thus promoting starch synthesis [93]. Z and ZR content was very significantly correlated with cell division rate of endosperm cells [94]. In our research, high ABA and ZR contents at early grain filling stage, which is beneficial to grain filling. The activity of CKs, together with auxin, is especially linked to growth promotion by cell division, development and differentiation [95], and auxin was reported to be responsible for the repression of CKs signaling through a feedback mechanism [96]. In this research, the trend of IAA content in caryopsis was 8 DAH < 12 DAH < 16 DAH, while trend of ZR content was 8 DAH > 12 DAH > 16 DAH pattern, thus the interaction between IAA and ZR might play important role in caryopsis development and chalkiness formation. In addition, at 8 DAH, ABA/IAA, ABA/ZR and IAA/ZR in high chalkiness caryopsis were higher than that in low chalkiness caryopsis, indicating the phytohormones contents and dynamic changes, the concentration gradient and the ratio of different phytohormones can regulate the expression of downstream genes, thus regulating grain development and chalkiness formation.

Overall, chalkiness formation is a dynamic process which is not only influenced by environmental factors, but also controlled by multiple genes, thus forming a complex regulatory network. Importantly, multiple phytohormonal signals are significantly different in chalkiness caryopsis, particularly those of ABA and auxin. Therefore, phytohormones play important roles through the interaction of multiple transcription factors and their downstream regulators during chalkiness formation.

## Conclusion

Rice chalkiness is a complex trait controlled by multiple genes. As an important rice quality trait, chalkiness not only affects the quality of rice, but also its price. How to effectively reduce the chalkiness of rice is an urgent task in rice production. In this study, X24 and its origin parents X11 and X7 were used as materials, and the phenotype, physiological and biochemical traits combined with transcriptome analysis in caryopsis from above cultivars were conducted to illustrate the dynamic formation and transcriptional regulation of rice chalkiness. The expression profile of genes related to starch/sucrose/protein metabolism and phytohormonal regulation, genes of transcription factors and PKs at 8 DAH, 12 DAH and 16 DAH showed that chalkiness formation is a dynamic process associated with multiple genes, forming a complex regulatory network in which phytohormones play a crucial role. Our results provided informative clues for illustrating the regulatory mechanisms of chalkiness formation in rice.

## Methods

### Plant material and growth conditions

Each of X24, X11 and X7 is pure line of *indica* rice with different chalkiness trait. X11 (pure line of *indica* rice with high-chalkiness) was bred by crossing Zhefu802 and Xiangzaoxian1 as origin parents, which was established until F_7_ generation by pedigree method [47, 97]. X7 (pure line of *indica* rice with low-chalkiness) was bred by crossing 81–280 and HA79317-4 as origin parents, which was established until F_5_ generation by pedigree method [47, 98]. X24 (pure line of *indica* rice with high-chalkiness) was bred by crossing X11 as female parent and X7 as male parent. It was established until F_6_ generation by pedigree method which superior individuals were selected from F_2_ generation [47, 99]. Obviously, the chalkiness of X24 is inherited from the female parent X11. The three cultivars of rice seeds were soaked in distilled water at 37 °C for 2 days, then the germinated seeds were sprinkled in wetted soil in natural condition. After 30 days, the seedlings were transferred to pots, 30 pots for each cultivar and 3 plants in each pot. Plants were grown in natural condition with daily average temperature 32 °C and night average temperature 24 °C.

### Measurement of chalkiness

Mature grains were harvested and dried at 37 °C for 5 days, and then measurement of chalky rice rate and chalkiness degree by micro-CT scanning and 3D reconstruction techniques [100]. Rice grains were embedded in Super Light Clay (ordered from Alibaba, China). Samples were loaded into SkyScan 1172 Micro-CT (Bruker, Belgium) and scanned in three dimensions. After then, a series of cross-section images captured by micro-CT system were used for the chalkiness quantification according to the principles of segmentation and 3D reconstruction in MIS. The volumes of chalkiness part were simultaneously calculated by MIS and then chalkiness degree was precisely quantified. After 3D reconstruction, the cross-section images became smooth plane. Then the smooth plane image was well distinguished, and the chalky rice rate was accurately quantified by Chalkiness 2.0 [101].

### Measurement of grain weight and size

Mature grains were harvested and dried at 37 °C for 5 days to measure grain weight and size. A total of 1000 grains were randomly counted from each sample and weighed to measure grain weight by electronic balance (AUX220, Shimadzu, Kyoto, Japan). The grain length and width from each sample were measured using Chalkiness 2.0 software [101].

### Scanning electron microscopy (SEM)

The caryopses collected at 8 DAH, 12 DAH, 16 DAH, 20 DAH and 24 DAH were examined by scanning electron microscopy (SEM). The caryopsis was dehulled and dried completely under low pressure, and then dehulled caryopsis was cut across the middle parts with a razor blade, the middle parts of caryopsis as samples and the cut surface coated with gold by an ion sputtering device (JFC-1600, JEOL, Tokyo, Japan). These samples were observed and photographed with a scanning electron microscope (JSM-6380LV, JEOL, Tokyo, Japan). SEM analysis was based on at least three biological replications of the mounted specimens. All procedures were carried out according to the manufacturer’s protocol.

### Measurement of starch and soluble protein contents in grains

Mature grains were sampled to measure the starch and soluble protein contents. Grains were dried at 37 °C and dehulled grains were ground to powder to analyze the starch content by the anthrone method [10]. 50 mg powder was added 2 mL hot distilled water, boiling water for 15 min, then added 200 μL perchloric acid (9.2 mol/L) to extract starch for 10 min, then volume to 10 mL, 4,000 g (5415R, Eppendorf, Germany) for 10 min, 200 μL supernatant was taken and 100 μL of 2% anthrone reagent (ethyl acetate dissolved) was added, and then added 1 mL H_2_SO_4_ slowly. 200 μL distilled water plus 100 μL of 2% anthrone reagent plus 1 mL H_2_SO_4_ as the control, boiling water for 10 min after complete reaction. 200 μL solution was detected at 620 nm using enzyme-labeled instrument (SPARK, TECAN, Switzerland). Starch solution of 0, 40, 80, 120, 160 and 200 μg/mL were used as the standard solution to make standard curve, and the content of starch in each sample was calculated according to the standard curve.

Soluble protein content was measured according to the Bradford assay [102]. 50 mg powder was added 1 mL of 0.25% NaOH and shocked extraction 30 min at 40 °C, then volume to 10 mL, 3,000 g for 10 min, 200 μL supernatant was taken and added 1 mL Coomassie Brilliant Blue G-250 (20 mg Coomassie Brilliant Blue powder was dissolved in 10 mL 90% ethanol with 20 mL 85% phosphoric acid and 10 mL distilled water), and then mixed well and set still for 2 min at room temperature, 200 μL distilled water was added 1 mL Coomassie Brilliant Blue G-250 as CK. 200 μL solution was detected at 595 nm using enzyme-labeled instrument. Bovine serum abumin solution of 0, 20, 40, 60, 80 and 100 μg/mL were used to make the standard curve, and the content of soluble protein in each sample was calculated according to the standard curve.

### Measurement of phytohormones contents in caryopsis

Caryopses from 1–4 stalks at the top of the panicle collected at 8, 12 and 16 DAH at 17:00—18:00 of X11, X7 and X24 and immediately wrapped in aluminum foil and frozen in liquid nitrogen, then stored at -80 °C until measurement of phytohormones contents. The caryopses from three panicles were used as one sample, all samples were measured in three biological replicates. Phytohormones were quantified by liquid chromatography-tandem mass spectrometry (8030 plus, Shimadzu, Kyoto, Japan) [103]. 100 mg caryopses were frozen by liquid nitrogen and well homogenized to powder using mortar. After addition of 1 mL of 80% methanol (HPLC grade, Merck, Darmstadt, Germany), homogenates were well mixed by ultrasonic bath (KQ3200E, Kunshan Ultrasonic, China) and kept at 4 °C for 12 h, 100 μL internal standards of deuterium-labeled phytohormones (^2^H_6_-ABA, ^2^H_5_-IAA, ^2^H_5_-ZR, Olchemim, Olomouc, Czech Republic) were added and mixed. After centrifugation at 12,000 g (5415R, Eppendorf, Germany) for 10 min, the supernatant was collected. Then after, 200 μL of 80% methanol (HPLC grade, Merck, Darmstadt, Germany) was added for suspension precipitation and kept at 4 °C for 4 h. After 12,000 g centrifugation for 10 min (4 °C), the supernatant was collected and merged with the first supernatant. The combined supernatant was dried in vacuumed concentrator (RCT 60, Jouan, France), dried extract was dissolved in 100 μL of 10% methanol and mixed. After 12,000 g centrifugation for 10 min (4 °C), 25 μL solution were then purified by liquid chromatography. Liquid chromatography was performed using UPLC BEH C_18_ column under column temperature of 40 °C. The mobile phase comprising solvent A (0.02% [v/v] aqueous acetic acid) and solvent B (100% [v/v] methanol) was employed in a gradient mode (time/A concentration/B concentration [min/%/%]: 0/90/10, 5/10/90, 6/10/90, and 6.1/80/20) at an eluent flow rate of 0.25 mL/min. The eluate was vacuumed to dryness again and dissolved in 20 μL of 10% methanol, the solution was then injected into the liquid chromatography-tandem mass spectrometry system. Collision energy of -16 eV and mass-to-charge ratio (m/z) of 174.2/130 for IAA, collision energy of 11 eV and m/z of 263.2/153.2 for ABA, and collision energy of -19 eV and m/z of 352.2/220.1 for ZR were employed. Experiments were repeated three times (three biological replicates) and each consisting of three replicates and similar results were obtained.

### Total RNA extraction, library preparation, and de novo sequencing

Caryopses from 1–4 stalks at the top of the panicle collected at 8 DAH, 12 DAH and 16 DAH on 17:00 to 18:00 of X11, X7 and X24 and immediately wrapped in aluminum foil and frozen in liquid nitrogen, then stored at -80 °C until use for transcriptome analysis. The caryopses from three panicles were used as one sample, all samples were collected in three biological replicates. The total RNA was extracted from caryopses samples using Trizol Reagent (Invitrogen, USA), and then RNA degradation and contamination was monitored on 1% agarose gels. RNA purity was checked using the NanoPhotometer®spectrophotometer (IMPLEN, CA, USA), RNA concentration was measured using Qubit®RNA Assay Kit in Qubit®2.0 Flurometer (Life Technologies, CA, USA). RNA integrity was assessed using the RNA Nano 6000 Assay Kit of the Agilent Bioanalyzer 2100 system (Agilent Technologies, CA, USA). A total of 1.5 μg RNA per sample was used as input material for the RNA sample preparations. Sequencing libraries were generated using NEBNext®UltraTMRNA Library Prep Kit for Illumina®(NEB, USA), and then the library preparations were sequenced on an Illumina Hiseq 4000 (Illumina, USA) and 150 bp paired-end reads were generated.

### De novo transcriptome assembly and functional annotation

Raw data were processed using NGS QC Toolkit [104], the reads containing ploy-N and the low quality reads were removed to obtain the clean reads. The read counts of each gene were obtained by Htseq-count [105] and the FPKM value of each gene was calculated using Cufflinks [106]. Differentially expression genes were identified using the DESeq (with replicates) functions estimateSizeFactors and nbinomTest [107]. *P*-value ≤ 0.05 and |Log_2_foldchange(FC)|≥ 1 determined as significantly differentially expression genes DEGs were selected and analyzed by GO (Gene Ontology) function and KEGG (Kyoto Encyclopedia of Genes and Genomes) pathway enrichment.

### Real-time PCR analysis

Total RNA was extracted from the caryopsis collected at 8 DAH, 12 DAH and 16 DAH of X11, X7 and X24. Total RNA was purified from gDNA contamination and reverse-transcribed to cDNA using TransScript One-Step gDNA Removal and cDNA Synthesis SuperMix (Transgen, Nanjing, China). A PCR program was performed on a CFX96-CFX384 Real-time PCR System (Bio-Rad, USA) in a final volume of 20 μL containing 1 μL of a 1/5 diluted cDNA template, 10 μL of the 2 × ChamQ SYBR Master Mix (Vazyme, China), and 0.5 μL (10 μM) of forward and reverse primers. Thermal cycler settings consisted of an initial hold at 95 °C for 30 s, then 45 cycles of 95 °C for 10 s, and 60 °C for 30 s. Three biological replications and three parallel reactions were performed. The actin gene of *indica* rice was used as internal standard, gene primers were shown in Table 3.

**Table 3:** The primer sequences for Real-time PCR

Gene	Primer sequences 5’-3’
*OsR498G0100430000.01*	F: GACGAGAGGAACAACTACCAG
	R: AGCAAGGTCTGGTTGTCC
*OsR498G0204716300.01*	F: AATCACCCCCAGATCCAGTA
	R: TGGCTCAGGTAGTTGGAGTAG
*OsR498G0917494500.01*	F: CTGATCCTCCTCGATTACGAC
	R: CTGATCCTCCTCGATTACGAC
*OsR498G0204791500.01*	F: AAGACGAAATCTACACCTGGAG
	R: CAAAACCTTCCTTCCTTGAGT
*PROLM9*	F: AAGTCTGGCAACAGCTCGC
	R: AGGGTGGTAATGGTACTGGGTG
*OsR498G0100472700.01*	F: ATAGACCAGGGCGGAAGGAA
	R: TGTTGGTCCCCACATTCAGG
*OsR498G0100734500.01*	F: CGTTGGGATCAGTTATGACATG
	R: CAAATTACTCCACCAGCAGTTG
*WRKY76*	F: GTGAAGAAGAAGGTGCAGC
	R: GAGGAGTTGATGGAGATGGAG
*OsYUCCA7*	F: TATGGAGGTGTCGCTGGA
	R: GAGGAGGACGAGGATCTTGTC
*OsARF10*	F: CCACCACCACCACCATG
	R: CAGGTCGGTGCTGATGATG
*OsSDR*	F: AAAGCTTGACATCTTGGTGAAC
	R: TTGGTGTTGAGGCATTTTACTG
*OsCPK21*	F: CAAACAAGTTCTTGAGCATCCA
	R: AGAGCCTTCTTCTTGAACTTGT
*Actin*	F: CTTCATAGGAATGGAAGCTGCGGGTA
	R: CGACCACCTTGATCTTCATGCTGCTA

### Statistical analysis

All experiments were conducted in at least three replicates. Statistical significance was evaluated using Microsoft Excel software. Values were expressed as means ± SE.

## Supplementary Information

**Additional file 1: Supplementary Fig. 1 **Analysis of DEG_HL_ in caryopsis. (A) DEG_HL_ obtained at 8 DAH, 12 DAH and 16 DAH, red column represents up-regulated of genes, blue column represents down-regulated of genes. (B) Comparison of Gene Ontology (GO) classifications of DEG_HL_ at 8 DAH, 12 DAH and 16 DAH. (C) KEGG pathway assignments of DEG_HL_ at 8 DAH, 12 DAH and 16 DAH, the top 10 categories are shown. DEG_HL_ were differentially expressed with statistical significance (*P*-value ≤ 0.05 and |Log_2_foldchange(FC)| ≥ 1).

## Data Availability

The datasets used and/or analyzed during the current study are available from the corresponding author on reasonable request.

## Abbreviations

SEM: Scanning electron microscope
PBs: Protein bodies
QTLs: Quantitative trait loci
PGWC: Percentage of grains with chalkiness
PCG: Percentage of chalky grains
ABA: Abscisic acid
IAA: Indole-3-acetic acid
CKs: Cytokinins
GAs: Gibberellins
Z: Zeatin
ZR: Zeatin riboside
JA: Jasmonates
ETH: Ethylene
SA: Salicylic acid
BRs: Brassionosteroids
DAH: Days after heading
DEGs: Differentially expressed genes
DEG_HL_: DEGs were only differentially expressed between high chalkiness and low chalkiness
TFs: Transcription factors
PKs: Protein kinases
GO: Gene Ontology
KEGG: Kyoto Encyclopedia of Genes and Genomes

## References

[1] Kim SS, Lee SE, Kim OW, Kim DC (2000). Physicochemical characteristics of chalky kernels and their effects on sensory quality of cooked rice. Cereal Chem.

[2] Ishimaru T, Horigane AK, Ida M, Iwasawa N, San-oh YA, Nakazono M, Nishizawa NK, Masumura T, Kondo M, Yoshida M (2009). Formation of grain chalkiness and changes in water distribution in developing rice caryopses grown under high-temperature stress. J Cereal Sci.

[3] Lin Z, Wang Z, Zhang X, Liu Z, Li G, Wang S, Ding Y (2017). Complementary proteome and transcriptome profiling in developing grains of a notched-belly rice mutant reveals key pathways involved in chalkiness formation. Plant Cell Physiol.

[4] Lin Z, Zheng D, Zhang X, Wang Z, Lei J, Liu Z, Li G, Wang S, Ding Y (2016). Chalky part differs in chemical composition from translucent part of japonica rice grains as revealed by a notched-belly mutant with white-belly. J Sci Food Agric.

[5] Zhou L, Chen L, Jiang L, Zhang W, Liu L, Liu X, Zhao Z, Liu S, Zhang L, Wang J (2009). Fine mapping of the grain chalkiness QTL qPGWC-7 in rice (*Oryza sativa* L.). Theoretical Appl Genet.

[6] Liu JF, Kui LM, Zhu ZF, Tan LB, Sun CQ (2007). Identification of QTLs associated with processing quality and appearance quality of common wild rice (*Oryza rufipogon* Griff.). J Agric Biotechnol.

[7] Song XJ, Huang W, Shi M, Zhu MZ, Lin HX (2007). A QTL for rice grain width and weight encodes a previously unknown RING-type E3 ubiquitin ligase. Nat Genet.

[8] Zhu A, Zhang Y, Zhang Z, Wang B, Xue P, Cao Y, Chen Y, Li Z, Liu Q, Cheng S (2018). Genetic dissection of qPCG1 for a quantitative trait locus for percentage of chalky grain in rice (*Oryza sativa* L.). Front Plant Sci.

[9] Wu S. Studies on the chalkiness related to physiological properties and QTL analysis of chalkiness trait in *indica* rice. Changsha: Hunan Agricultural University; 2002.

[10] Li Y, Fan C, Xing Y, Yun P, Luo L, Yan B, Peng B, Xie W, Wang G, Li X (2014). *Chalk5* encodes a vacuolar H^+^-translocating pyrophosphatase influencing grain chalkiness in rice. Nat Genet.

[11] Zhao X, Daygon VD, McNally KL, Hamilton RS, Xie F, Reinke RF, Fitzgerald MA (2015). Identification of stable QTLs causing chalk in rice grains in nine environments. Theor Appl Genet.

[12] Umemoto T, Terashima K, Nakamura Y, Satoh H (2010). Differences in amylopectin structure between two rice varieties in relation to the effects of temperature during grain-filling. Starch-Stärke.

[13] Asaoka MM, Okuno K, Sugimoto Y, Kawakami J, Fuwa H (2010). Effect of environmental temperature during development of rice plants on some properties of endosperm starch. Starch-Stärke.

[14] Hakata M, Kuroda M, Miyashita T, Yamaguchi T, Kojima M, Sakakibara H, Mitsui T, Yamakawa H (2012). Suppression of alpha-amylase genes improves quality of rice grain ripened under high temperature. Plant Biotechnol J.

[15] Lin SK, Chang MC, Tsai YG, Lur HS (2005). Proteomic analysis of the expression of proteins related to rice quality during caryopsis development and the effect of high temperature on expression. Proteomics.

[16] Lunde C, Zygadlo A, Simonsen HT, Nielsen PL, Haldrup A (2010). Sulfur starvation in rice: the effect on photosynthesis, carbohydrate metabolism, and oxidative stress protective pathways. Physiol Plant.

[17] Jiang W, Zhou M, Li T (2002). Anatomical study on the chalkiness formation of early indica rice. J Zhejiang Univ.

[18] Yamakawa H, Hirose T, Kuroda M, Yamaguchi T (2007). Comprehensive expression profiling of rice grain filling-related genes under high temperature using DNA microarray. Plant Physiol.

[19] Maccarrone M, Melino G, Finazzi-Agro A (2001). Lipoxygenases and their involvement in programmed cell death. Cell Death Differ.

[20] Gorantla M, Babu P, Lachagari V, Reddy A, Wusirika R, Bennetzen J, Reddy A (2007). Identification of stress-responsive genes in an *indica* rice (*Oryza sativa* L.) using ESTs generated from drought-stressed seedlings. J Exper Botany.

[21] Cheng C, Yun K, Ressom H, Mohanty B, Bajic V, Jia Y, Yun S (2007). de los Reyes B: An early response regulatory cluster induced by low temperature and hydrogen peroxide in seedlings of chilling-tolerant japonica rice. BMC Genomics.

[22] Mikami I, Aikawa M, Hirano HY, Sano Y (1999). Altered tissue-specific expression at the *Wx* gene of the opaque mutants in rice. Euphytica.

[23] Fujita N, Yoshida M, Kondo T, Saito K, Utsumi Y, Tokunaga T, Nishi A, Satoh H, Park JH, Jane JL (2007). Characterization of SSIIIa-deficient mutants of rice: the function of SSIIIa and pleiotropic effects by SSIIIa deficiency in the rice endosperm. Plant Physiol.

[24] Nishi A, Nakamura Y, Tanaka N, Satoh H (2001). Biochemical and genetic analysis of the effects of amylose-extender mutation in rice endosperm. Plant Physiol.

[25] Zhang D, Wu J, Zhang Y, Shi C (2012). Phenotypic and candidate gene analysis of a new floury endosperm mutant *(osagpl2-3*) in rice. Plant Mol Biol Report.

[26] Kang HG, Park S, Matsuoka M, An G (2005). White-core endosperm floury endosperm-4 in rice is generated by knockout mutations in the C4-type pyruvate orthophosphate dikinase gene (*OsPPDKB*). Plant J.

[27] She KC, Kusano H, Koizumi K, Yamakawa H, Hakata M, Imamura T, Fukuda M, Naito N, Tsurumaki Y, Yaeshima M (2010). A novel factor *FLOURY ENDOSPERM2* is involved in regulation of rice grain size and starch quality. Plant Cell.

[28] Peng C, Wang Y, Liu F, Ren Y, Zhou K, Lv J, Zheng M, Zhao S, Zhang L, Wang C (2014). *FLOURY ENDOSPERM6* encodes a CBM48 domain-containing protein involved in compound granule formation and starch synthesis in rice endosperm. Plant J.

[29] Satoh H, Shibahara K, Tokunaga T, Nishi A, Tasaki M, Hwang SK, Okita TW, Kaneko N, Fujita N, Yoshida M (2008). Mutation of the plastidial alpha-glucan phosphorylase gene in rice affects the synthesis and structure of starch in the endosperm. Plant Cell.

[30] Chao SF, Cai YC, Feng BB, Jiao GA, Sheng ZH, Luo J, Tang SQ, Wang JL, Song HP, Wei XJ (2019). Editing of rice isoamylase gene *ISA1* provides insights into its function in starch formation. Rice Sci.

[31] Lin CJ, Li CY, Lin SK, Yang FH, Huang JJ, Liu YH, Lur HS (2014). Influence of high temperature during grain filling on the accumulation of storage proteins and grain quality in rice (*Oryza sativa* L.). J Agric Food Chem.

[32] Yamakawa H, Hakata M (2010). Atlas of rice grain filling-related metabolism under high temperature: joint analysis of metabolome and transcriptome demonstrated inhibition of starch accumulation and induction of amino acid accumulation. Plant Cell Physiol.

[33] Bai AN, Lu XD, Li DQ, Liu JX, Liu CM (2016). NF-YB1-regulated expression of sucrose transporters in aleurone facilitates sugar loading to rice endosperm. Cell Res.

[34] Xu JJ, Zhang XF, Xue HW (2016). Rice aleurone layer specific OsNF-YB1 regulates grain filling and endosperm development by interacting with an ERF transcription factor. J Exp Bot.

[35] Fu FF, Xue HW (2010). Coexpression analysis identifies Rice Starch Regulator1, a rice AP2/EREBP family transcription factor, as a novel rice starch biosynthesis regulator. Plant Physiol.

[36] Wang JC, Xu H, Zhu Y, Liu QQ, Cai XL (2013). OsbZIP58, a basic leucine zipper transcription factor, regulates starch biosynthesis in rice endosperm. J Exp Bot.

[37] Jiang JZ, Kuo CH, Chen BH, Chen MK, Lin CS, Ho SL (2018). Effects of *OsCDPK1* on the structure and physicochemical properties of starch in developing rice seeds. Int J Mol Sci.

[38] Kim YJ, Yeu SY, Park BS, Koh HJ, Song JT, Seo HS (2012). Protein disulfide isomerase-like protein 1–1 controls endosperm development through regulation of the amount and composition of seed proteins in rice. Plos One.

[39] Wakasa Y, Yasuda H, Oono Y, Kawakatsu T, Hirose S, Takahashi H, Hayashi S, Yang L, Takaiwa F (2011). Expression of ER quality control-related genes in response to changes in BiP1 levels in developing rice endosperm. Plant J.

[40] Yasuda H, Hirose S, Kawakatsu T, Wakasa Y, Takaiwa F (2009). Overexpression of BiP has inhibitory effects on the accumulation of seed storage proteins in endosperm cells of rice. Plant Cell Physiol.

[41] Liu F, Ren Y, Wang Y, Peng C, Zhou K, Lv J, Guo X, Zhang X, Zhong M, Zhao S (2013). OsVPS9A functions cooperatively with OsRAB5A to regulate post-Golgi dense vesicle-mediated storage protein trafficking to the protein storage vacuole in rice endosperm cells. Mol Plant.

[42] Fukuda M, Wen L, Satoh-Cruz M, Kawagoe Y, Nagamura Y, Okita TW, Washida H, Sugino A, Ishino S, Ishino Y (2013). A guanine nucleotide exchange factor for Rab5 proteins is essential for intracellular transport of the proglutelin from the golgi apparatus to the protein storage vacuole in rice endosperm. Plant Physiol.

[43] Ren Y, Wang Y, Liu F, Zhou K, Ding Y, Zhou F, Wang Y, Liu K, Gan L, Ma W (2014). *GLUTELIN PRECURSOR ACCUMULATION3* encodes a regulator of post-golgi vesicular traffic essential for vacuolar protein sorting in rice endosperm. Plant Cell.

[44] Zhang XF, Tong JH, Bai AN, Liu CM, Xiao LT, Xue HW (2020). Phytohormone dynamics in developing endosperm influence rice grain shape and quality. J Integr Plant Biol.

[45] Kabir MH, Liu Q, Su Y, Huang ZG, Xiao LT (2017). Dynamics of phytohormones and their relationship with chalkness of early *indica* rice under different post-anthesis temperature regimes. Bangladesh J Agric Res.

[46] Yang JC, Wang ZQ, Zhu QS, Su BL (1999). Regulation of ABA and GA to the grain filling of rice. Acta Agron Sin.

[47] China Rice Date Centre. In. http://www.ricedata.cn/.

[48] Duan M, Sun SSM (2005). Profiling the expression of genes controlling rice grain quality. Plant Mol Biol.

[49] Kyndt T, Denil S, Haegeman A, Trooskens G, Meyer D, Criekinge V, Gheysen G (2012). Transcriptome analysis of rice mature root tissue and root tips in early development by massive parallel sequencing. J Exp Bot.

[50] Wang D, Pan Y, Zhao X, Zhu L, Li Z (2011). Genome-wide temporal-spatial gene expression profiling of drought responsiveness in rice. BMC Genomics.

[51] Zhu N, Cheng S, Liu X, Du H, Dai M, Zhou DX, Yang W, Zhao Y (2015). The R2R3-type MYB gene OsMYB91 has a function in coordinating plant growth and salt stress tolerance in rice. Plant Sci.

[52] Yang H, Xiong H, Li J, Liu P, Duan J, Zhao Y, Guo X, Li Y, Zhang H, Ali J (2014). Overexpression of OsMYB48–1, a Novel MYB-related transcription factor, enhances drought and salinity tolerance in rice. Plos One.

[53] Serra TS, Figueiredo DD, Cordeiro AM, Almeida DM, Lourenco T, Abreu IA, Sebastian A, Fernandes L, Contreras-Moreira B, Oliveira MM (2013). OsRMC, a negative regulator of salt stress response in rice, is regulated by two AP2/ERF transcription factors. Plant Mol Biol.

[54] Eulgem T, Somssich IE (2007). Networks of WRKY transcription factors in defense signaling. Curr Opin Plant Biol.

[55] Pandey SP, Somssich IE (2009). The Role of WRKY Transcription Factors in Plant Immunity. Plant Physiol.

[56] Peng Y, Bartley LE, Canlas P, Ronald PC (2010). OsWRKY IIa transcription factors modulate rice innate immunity. Rice.

[57] Koo SC, Moon BC, Kim JK, Kim CY, Sung SJ, Kim MC, Cho MJ, Cheong YH (2009). OsBWMK1 mediates SA-dependent defense responses by activating the transcription factor OsWRKY33. Biochem Biophys Res Commun.

[58] Yu S, Shaojuan J, Diqiu YU (2009). Overexpression of the stress-induced OsWRKY08 improves osmotic stress tolerance in *Arabidopsis*. Chin Sci Bull.

[59] Naoki Y, Yuko S, Shigeru T, Tetsuya C, Takafumi S, Kazunori O, Hisakazu Y, Masaki S, Shoji S, Hiroshi T (2013). WRKY76 is a rice transcriptional repressor playing opposite roles in blast disease resistance and cold stress tolerance. J Exp Bot.

[60] Hudson D, Guevara DR, Hand AJ, Xu Z, Hao L, Chen X, Zhu T, Bi YM, Rothstein SJ (2013). Rice cytokinin GATA transcription factor1 regulates chloroplast development and plant architecture. Plant Physiol.

[61] Laurie S, Halford NG (2001). The role of protein kinases in the regulation of plant growth and development. Plant Growth Regul.

[62] Galvan-Ampudia CS, Offringa R (2007). Plant evolution: AGC kinases tell the auxin tale. Trends Plant Sci.

[63] Morris ER, Walker JC (2003). Receptor-like protein kinases: the keys to response. Curr Opin Plant Biol.

[64] Li JM, Chory J (1997). A putative leucine-rich repeat receptor kinase involved in brassinosteroid signal transduction. Cell.

[65] Li J, Wen J, Lease KA, Doke JT, Tax FE, Walker JC (2002). BAK1, an Arabidopsis LRR receptor-like protein kinase, interacts with BRI1 and modulates brassinosteroid signaling. Cell.

[66] Cheng Y, Qi Y, Zhu Q, Chen X, Zhang W (2010). New changes in the plasma-membrane-associated proteome of rice roots under salt stress. Proteomics.

[67] Asano T, Hayashi N, Kikuchi S, Ohsugi R (2012). CDPK-mediated abiotic stress signaling. Plant Signal Behav.

[68] Manuka R, Karle SB, Kumar K (2019). OsWNK9 mitigates salt and drought stress effects through induced antioxidant systems in *Arabidopsis*. Plant Physiol Rep.

[69] Asano T, Hakata M, Nakamura H, Aoki N, Komatsu S, Ichikawa H, Hirochika H, Ohsugi R (2011). Functional characterisation of OsCPK21, a calcium-dependent protein kinase that confers salt tolerance in rice. Plant Mol Biol.

[70] Ohdan T, Francisco PB, Sawada T, Hirose T, Terao T, Satoh H, Nakamura Y (2005). Expression profiling of genes involved in starch synthesis in sink and source organs of rice. J Exp Bot.

[71] Han X, Wang Y, Liu X, Jiang L, Ren Y, Liu F, Peng C, Li J, Jin X, Wu F (2012). The failure to express a protein disulphide isomerase-like protein results in a floury endosperm and an endoplasmic reticulum stress response in rice. J Exp Bot.

[72] Ball S, Morell M (2003). From bacterial glycogen to starch: understanding the biogenesis of the plant starch granule. Annu Rev Plant Biol.

[73] Patrick JW, Offler CE (2001). Compartmentation of transport and transfer events in developing seeds. J Exp Bot.

[74] Zhang WH, Zhou Y, Dibley KE, Tyerman SD, Furbank RT, Patrick JW (2007). Review: Nutrient loading of developing seeds. Funct Plant Biol.

[75] Woo MO, Ham TH, Ji HS, Choi MS, Koh HJ (2008). Inactivation of the *UGPase1* gene causes genic male sterility and endosperm chalkiness in rice (*Oryza sativa* L.). Plant J.

[76] Zhang L, Ren Y, Lu B, Yang C, Feng Z, Liu Z, Chen J, Ma W, Wang Y, Yu X (2016). *FLOURY ENDOSPERM7* encodes a regulator of starch synthesis and amyloplast development essential for peripheral endosperm development in rice. J Exp Bot.

[77] Wu M, Ren Y, Cai M, Wang Y, Zhu S, Zhu J, Hao Y, Teng X, Zhu X, Jing R (2019). Rice *FLOURY ENDOSPERM10* encodes a pentatricopeptide repeat protein that is essential for the trans-splicing of mitochondrial nad1 intron 1 and endosperm development. New Phytol.

[78] Teng X, Zhong M, Zhu X, Wang C, Ren Y, Wang Y, Zhang H, Jiang L, Wang D, Hao Y (2019). *FLOURY ENDOSPERM16* encoding a NAD-dependent cytosolic malate dehydrogenase plays an important role in starch synthesis and seed development in rice. Plant Biotechnol J.

[79] Matsushima R, Maekawa M, Kusano M, Kondo H, Fujita N, Kawagoe Y, Sakamoto W (2014). Amyloplast-localized SUBSTANDARD STARCH GRAIN4 protein influences the size of starch grains in rice endosperm. Plant Physiol.

[80] Matsushima R, Maekawa M, Kusano M, Tomita K, Kondo H, Nishimura H, Crofts N, Fujita N, Sakamoto W (2016). Amyloplast membrane protein SUBSTANDARD STARCH GRAIN6 controls starch grain size in rice endosperm. Plant Physiol.

[81] Tanabe S, Ashikari M, Fujioka S, Takatsuto S, Yoshida S, Yano M, Yoshimura A, Kitano H, Matsuoka M, Fujisawa Y (2005). A novel cytochrome P450 is implicated in brassinosteroid biosynthesis via the characterization of a rice dwarf mutant, *dwarf11*, with reduced seed length. Plant Cell.

[82] Tong H, Liu L, Jin Y, Du L, Yin Y, Qian Q, Zhu L, Chu C (2012). DWARF and LOW-TILLERING acts as a direct downstream target of a GSK3/SHAGGY-like kinase to mediate brassinosteroid responses in rice. Plant Cell.

[83] Yin LL, Xue HW (2012). The MADS29 transcription factor regulates the degradation of the nucellus and the nucellar projection during rice seed development. Plant Cell.

[84] Ishimaru K, Hirotsu N, Madoka Y, Murakami N, Hara N, Onodera H, Kashiwagi T, Ujiie K, Shimizu B, Onishi A (2013). Loss of function of the IAA-glucose hydrolase gene *TGW6* enhances rice grain weight and increases yield. Nat Genet.

[85] Zuo J, Li J (2014). Molecular genetic dissection of quantitative trait loci regulating rice grain size. Annu Rev Genet.

[86] Bello BK, Hou Y, Zhao J, Jiao G, Wu Y, Li Z, Wang Y, Tong X, Wang W, Yuan W (2019). NF-YB1-YC12-bHLH144 complex directly activates *Wx* to regulate grain quality in rice (*Oryza sativa* L.). Plant Biotechnol J.

[87] Menkens AE (1995). The G-box: a ubiquitous regulatory DNA element in plants bound by the GBF family of bZIP proteins. Trends Biochem Sci.

[88] Wu J, Zhu C, Pang J, Zhang X, Yang C, Xia G, Tian Y, He C (2014). OsLOL1, a C2C2-type zinc finger protein, interacts with OsbZIP58 to promote seed germination through the modulation of gibberellin biosynthesis in *Oryza sativa*. Plant J.

[89] Xiong Y, Ren Y, Li W, Wu F, Yang W, Huang X, Yao J (2019). NF-YC12 is a key multi-functional regulator of accumulation of seed storage substances in rice. J Exp Bot.

[90] Wang PC, Hsu CC, Du YY, Zhu PP, Zhao CZ, Fu X, Zhang CG, Paez J, Macho AP, Taob WA (2020). Mapping proteome-wide targets of protein kinases in plant stress responses. Proc Natl Acad Sci.

[91] Ho SL, Huang LF, Lu CA, He SL, Wang CC, Yu SP, Chen J, Yu SM (2013). Sugar starvation- and GA-inducible calcium-dependent protein kinase 1 feedback regulates GA biosynthesis and activates a 14-3-3 protein to confer drought tolerance in rice seedlings. Plant Mol Biol.

[92] Locascio A, Roig-Villanova I, Bernardi J, Varotto S (2014). Current perspectives on the hormonal control of seed development in Arabidopsis and maize: a focus on auxin. Front Plant Sci.

[93] Rook F, Corke F, Card R, Munz G, Smith C, Bevan MW (2010). Impaired sucrose-induction mutants reveal the modulation of sugar-induced starch biosynthetic gene expression by abscisic acid signalling. Plant J.

[94] Yang ZC, Zhang JH, Wang ZQ, Zhu QS (2003). Hormones in the grains in relation to sink strength and postanthesis development of spikelets in rice. Plant Growth Regul.

[95] Vanstraelen M, Benkova E (2012). Hormonal interactions in the regulation of plant development. Curr Opin Plant Biol.

[96] Chen J, Lausser A, Dresselhaus T (2014). Hormonal responses during early embryogenesis in maize. Biochem Soc Trans.

[97] Zeng DH, Peng MY, Li MY, Zheng RF, Jiang XC (1991). Study on Breeding of Middle-maturing New Early *Indica* Variety Xiangzaoxian11. Hunan Agricultural Sciences.

[98] Yang YZ (1990). New Early *Indica* Variety Xiangzaoxian7. Crops.

[99] Zeng DH, Peng MY, Li MY, Zheng RF, Jiang XC, Sun GH (1997). Study on Breeding of Middle-maturing New Early *Indica* Variety Xiangzaoxian24. Hunan Agric Sci.

[100] Su Y, Xiao LT (2020). 3D Visualization and Volume-Based Quantification of Rice Chalkiness In Vivo by Using High Resolution Micro-CT. Rice.

[101] Xiao LT, Lin WH, Li DH, Hong B (2001). A method to measure the rice kernel chalkiness objectively. Chin Rice Res Newsl.

[102] Bradford (1976). A rapid and sensitive method for the quantitation of microgam quantities of protein utilizing the principle of protein-dye binding. Analytical Biochem.

[103] Zhou LJ, Xiao LT, Xue HW (2017). Dynamic cytology and transcriptional regulation of rice lamina joint development. Plant Physiol.

[104] Patel RK, Mukesh J, Liu Z (2012). NGS QC Toolkit: A toolkit for quality control of next generation sequencing data. Plos One.

[105] Simon A, Theodor PP, Wolfgang H (2015). HTSeq—a Python framework to work with high-throughput sequencing data. Bioinformatics.

[106] Trapnell C, Roberts A, Goff L, Pertea G, Kim D, Kelley DR, Pimentel H, Salzberg SL, Rinn JL, Pachter L (2012). Differential gene and transcript expression analysis of RNA-seq experiments with topHat and cufflinks. Nat Protoc.

[107] Anders S HW: Differential expression of RNA-Seq data at the gene level–the DESeq package. In. http://www.bioconductor.org/packages/release/bioc/vignettes/DESeq/inst/doc/DESeq; 2016.

